# DARTpaths, an *in silico* platform to investigate molecular mechanisms of compounds

**DOI:** 10.1093/bioinformatics/btac767

**Published:** 2022-12-08

**Authors:** Diksha Bhalla, Marvin N Steijaert, Eefje S Poppelaars, Marc Teunis, Monique van der Voet, Marie Corradi, Elisabeth Dévière, Luke Noothout, Wilco Tomassen, Martijn Rooseboom, Richard A Currie, Cyrille Krul, Raymond Pieters, Vera van Noort, Marjolein Wildwater

**Affiliations:** Centre of Microbial and Plant Genetics, Faculty of Bioscience Engineering, KU Leuven, Leuven 3001, Belgium; Open Analytics, Antwerp 2600, Belgium; Vivaltes, Bunnik 3981 LA, The Netherlands; Innovative Testing in Life Sciences & Chemistry, Utrecht University of Applied Sciences, Utrecht 3584 CH, The Netherlands; Vivaltes, Bunnik 3981 LA, The Netherlands; Innovative Testing in Life Sciences & Chemistry, Utrecht University of Applied Sciences, Utrecht 3584 CH, The Netherlands; Open Analytics, Antwerp 2600, Belgium; CLEVER FRANKE, Utrecht 3512 CC, The Netherlands; CLEVER FRANKE, Utrecht 3512 CC, The Netherlands; Toxicology Group, Shell Global Solutions International B.V., The Hague 2596 HR, the Netherlands; Predictive and Computational Toxicology, Syngenta, Jealott's Hill International Research Centre, Bracknell, Berkshire RG42 6EY, UK; Innovative Testing in Life Sciences & Chemistry, Utrecht University of Applied Sciences, Utrecht 3584 CH, The Netherlands; Innovative Testing in Life Sciences & Chemistry, Utrecht University of Applied Sciences, Utrecht 3584 CH, The Netherlands; Utrecht University, Institute for Risk Assessment Sciences, Utrecht 3584 CM, The Netherlands; Centre of Microbial and Plant Genetics, Faculty of Bioscience Engineering, KU Leuven, Leuven 3001, Belgium; Leiden University, Institute of Biology Leiden, Leiden 2333 BE, The Netherlands; Vivaltes, Bunnik 3981 LA, The Netherlands

## Abstract

**Summary:**

Xpaths is a collection of algorithms that allow for the prediction of compound-induced molecular mechanisms of action by integrating phenotypic endpoints of different species; and proposes follow-up tests for model organisms to validate these pathway predictions. The Xpaths algorithms are applied to predict developmental and reproductive toxicity (DART) and implemented into an *in silico* platform, called DARTpaths.

**Availability and implementation:**

All code is available on GitHub https://github.com/Xpaths/dartpaths-app under Apache license 2.0, detailed overview with demo is available at https://www.vivaltes.com/dartpaths/.

**Supplementary information:**

Supplementary data are available at *Bioinformatics* online.

## 1 Introduction

Regulatory compliance to address the human health risks of chemicals commonly still requires both rodents and non-rodents as test species. In addition to these species not always being reliable due to species-specific differences, they are not ethically sustainable in the current century. For this reason, a broad portfolio of new approach methods (NAMs) have been established over the last years, such as a wide diversity of cell- and organoid-based protocols, assays that use small organisms like *Caenorhabditis elegans* (*C.elegans-nematodes*), *Danio rerio* (zebrafish) or *Dictyostelium discoideum* (*D.discoideum-slime mold*), as well as several *in silico* platforms.

The largest challenge remains how to integrate and combine the study data of all these different screening platforms into a single framework. Such a framework connects and enhances the predictivity of the scientific and regulatory framework for hazard and risk assessment. With Xpaths, we created a data model and algorithms for interspecies integration of test outcomes. The resulting cross-species profiles of compound mechanistic activity can support regulatory acceptance and approval of NAMs. To predict effects in higher organisms like humans, compound-affected evolutionary conserved molecular pathways are identified using multiple test methodologies. The goal is to test compound-induced effects in multiple (NAM and/or other) test methodologies, identify evolutionary conserved mechanistic pathways and combine this information to predict the effect of the same compound in humans and other mammals. Those pathways form a key to identify the most relevant and conserved toxicity endpoint effects. The ontology-linked approach of Xpaths allows translation of toxicity (phenotypic) endpoints of different studies in different species, into a mechanistical knowledge-based prediction output, using statistical pathway ranking. Data from a broad set of cell-based assays as well as six major model organisms: *D.discoideum* (slime mold/social amoebe)*, C.elegans* (nematode), *Drosophila melanogaster* (fruitfly), *D.rerio* (zebrafish), *Mus musculus* (mouse) and *Homo sapiens* (human) form the foundation of the Xpaths model. In addition, the model can predict which assays in *C.elegans, D.melanogaster* or *D.rerio* are relevant for further validation of predictions of possible (adverse outcome) mechanisms. With Xpaths, we aimed to create a single data model for inter-species connection of test outcomes, with the overall goal to improve the reliability of compound effect predictions.

## 2 Materials and methods

**Fig. 1.**
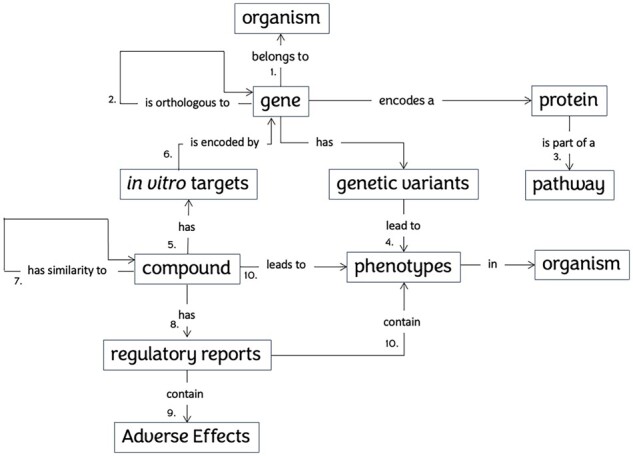
Semantic data model of Xpaths. Connection of data types in the DARTpaths application. 1. Gene-organism from ENSEMBL 2. Orthology from ENSEMBL Compara 3. Gene—Protein—pathway from Reactome 4. Genetic variants to phenotypes from organism specific databases 5. Compound target *in vitro* data from EPA 6. *In vitro* target names to genes from ENSEMBL 7. Computed fingerprint similarities 8. Regulatory reports from QSAR toolbox 9. Toxicity conclusion extracted with Xpaths scripts 10. Text mining of regulatory reports and literature (mammalian), compound-phenotype links from model organism databases. The paths between phenotypes/endpoint effects in mammals, pathways/mechanisms, phenotypes/endpoint effects in New Alternative Methods and phenotypes/endpoint effects in humans are calculated by Xpaths as an alternative to the traditional paths between phenotypes/endpoint effects in mammals and those in humans

The Xpaths algorithms are written in R (https://www.R-project.org/) and Python. Data are made interoperable using standard vocabularies (Fig. 1) and are integrated from databases that contain regulatory study information (QSAR toolbox), model organism-specific data sources (WormBase, DictyBase, FlyBase, Zebrafish Information Network and Mouse Genome Informatics) and biology databases (Reactome, ENSEMBL). Xpaths’ pathways are based on Reactome (Jassal *et al.*, 2019) and orthology information is retrieved from ENSEMBL compara (Herrero *et al.*, 2016). The sources (Bult *et al.*, 2019; Chisholm *et al.*, 2006; Harris *et al.*, 2019; Larkin *et al.*, 2021; Ruzicka *et al.*, 2018) and the process of implementing the backend database for each of the eight organisms are described in Supplementary material.

### 2.1 Phenotype pathway enrichment analysis

The phenotype pathway enrichment analysis (PPEA) module takes as input a list of phenotype ontology identifiers observed in model organisms after exposure to a chemical (Fig. 1). The algorithm first retrieves genes associated to the phenotypes and uses an area under the curve algorithm to find the best matching pathways, similar to a gene set enrichment analysis (Subramanian *et al.*, 2005). For ease of interpretation of the AUC score, *P*-values are estimated by Monte Carlo random sampling. Cross-species *P*-values are combined using the harmonic mean. The main difference between the Xpaths PPEA algorithm and already existing phenotype enrichment tools, is that those tools typically use a list of genes as a starting point for the analysis. In Xpaths, the starting point is a list of observed phenotypes in one or multiple species and the output integrates this information with the conservation of pathways of the test species to human pathways. Details of the PPEA are discussed in Supplementary material.

### 2.2 Phenotype prediction from evolutionary conserved pathway data

Phenotype pathway enrichment analysis identifies phenotypes that are enriched for gene sets comprising of genes in a human pathway and their orthologs in model species. Expected phenotypes in model organism can be predicted. Ortholog data were retrieved from ENSEMBL Compara (Herrero *et al.*, 2016). The *P*-values in the phenotype enrichment module are computed from a hypergeometric distribution and are subsequentially adjusted by FDR-correction (Benjamini and Hochberg, 1995). Details of the phenotype prediction algorithms are discussed in the Supplementary material.

## 3 Results

Xpaths is designed for cross-species connection of test outcomes to increase reliability in compound effect prediction. It is designed to be easy to use and is based on FAIR principles (i.e. findable, accessible, interoperable and reusable).

The data model can be reused for other comparative pathways projects by providing all extraction, transformation and loading scripts and enrichment algorithms via a GitHub repository. At the same time the user-friendly interface makes the tool widely applicable. Future work might be able to make use of the Xpaths algorithms as a general data model. Currently, the Xpaths algorithms have been implemented in the DARTpaths platform.

### 3.1 DARTpaths

DARTpaths is designed as an *in silico* platform to predict developmental and reproductive toxicity (DART). The DARTpaths platform is created as an R Shiny app. All code is available on GitHub.

#### 3.1.1 Substance exploration

Users can start by searching for a single chemical or a complex substance (i.e. UVCB), either by name or by identifier (CAS, EC and SMILES). Endpoint data from different test methodologies (cell-based assays, as well as phenotype observations from rat, rabbit, *D.discoideum, C.elegans, D.melanogaster*, *D.rerio* and *M.musculus*) is integrated to predict which pathways are potentially affected by the chemical. An integrated *P*-value indicates the strength of evidence for that pathway. For example, when searching with DiEthylStilbestrol (DES), the top pathways predicted include: ‘Metbolism of steroids’; this is supported by two *in vitro* hits CYP19A1 and TSPO- these targets are both part of the pathway. In addition, the specific phenotypes caused by genetic variants in this pathway resemble the phenotypes induced by DES in zebrafish, nematodes (3 out of 8 overlap) and mammals (10 out of 20 overlap). DES, has indeed been shown to interfere with this pathway (see Supplementary materials for detailed information). Pathways are ranked based on the *P*-value and can be filtered by type of evidence: non-mammalian phenotypes, mammalian phenotypes, *in vitro* targets, all evidence, any type of evidence. Furthermore, when selecting a pathway, the user sees the number of genes in this pathway in humans, cross-species conservation of genes between humans and each model species, and phenotypes linked to this pathway categorized by evidence type. Detailed information is available for similar substances to this chemical, including additional information to assist in selecting related chemicals, such as *in vitro* test data of this chemical (with assay type and link to the source database), phenotypes and regulatory information about the chemical.

#### 3.1.2 Pathway exploration

Users can also start by selecting a pathway. Pathway exploration shows the type of orthology relations (one-to-one, one-to-many and many-to-many) for each organism for all genes in this human pathway. For example, it is known that dioxin-like chemicals bind to the aryl hydrocarbon receptor (AHR). By starting from the pathway ‘Aryl Hydrocarbon Receptor Signaling’, a user can see which non-mammalian model organisms contain this pathway and might be used to study the toxicity of dioxin-like compounds. The AHR pathway is highly conserved in nematodes and thus, toxicity of these type of compounds can be studied in nematodes. This is particularly useful for testing mixtures as reported by EPA (Krewski *et al.*, 2010). Finally, it can be used to predict phenotypic endpoints in model species *C*.*elegans, D.melanogaster* (fruit fly) and *D.rerio* (zebrafish). The phenotype ‘neurite connectivity defective’ is observed in genetic variants of AHR in nematodes. One might expect dioxin-like chemicals to induce similar phenotypes. In this way, one can further validate pathway predictions.

#### 3.1.3 Database

Currently, the DARTpaths database contains information on DART-related phenotypic endpoints for model organisms (Table 1) and *in vitro* assays. Compound-phenotype connections are obtained from model organism databases, where they are described using structured vocabularies. For mammalian data, compound-phenotype links are only available in text format. To add data on phenotypic endpoints of mammals from scientific articles and study reports, we have developed text mining algorithms (to extract phenotypes, compounds, organisms, doses, exposure routes, parent versus offspring and *in vitro* versus *in vivo*) using natural language processing with SpaCy (spacy.io; Neumann *et al.*, 2019) and mammalian phenotype ontology (Smith and Eppig, 2009) linking with PhenoTagger (Luo *et al.*, 2021). This approach was applied for two compounds (DES and Thalidomide; Table 1). This text mining can be used on demand to further populate the database and thereby improve the pathway predictions.

**Table 1.** Compound–phenotype connections in the DARTpaths application

	Compound–phenotype connections	Unique compounds with phenotypes
Zebrafish (Zebrafish phenotype ontology)	10 244	1016
Nematode (Wormbase phenotype)	3213	278
Mammals (Mammalian phenotype)	42	2

## Supplementary Material

btac767_Supplementary_Data

## Data Availability

LOA substance categories, mammalian phenotypes and fine-grained orthology are provided in the dartpaths repository on GitHub. The rest of underlying data belong to third parties. The GitHub repository provides scripts for data download, transformation and loading into the database. Some data needs to be downloaded manually after agreement with terms and conditions. All instructions are provided in the folder ETL.
