# Supplementary material for: DARTpaths, an in silico platform to investigate molecular mechanisms of compounds
Source: Bioinformatics. 2022 Dec 8;39(1):btac767. doi: 10.1093/bioinformatics/btac767 (PMC9825785; doi:10.1093/bioinformatics/btac767)
Supplement: btac767_Supplementary_Data [file btac767_supplementary_data.docx]

DARTpaths, an *in silico* platform to investigate molecular mechanisms of compounds

Supplementary materials

Diksha Bhalla^1*^, Marvin N. Steijaert^2*^, Eefje S. Poppelaars^3*^, Marc Teunis^4*^, Monique van der Voet^3^, Marie Corradi^4^, Elisabeth Dévière^2^, Luke Noothout^5^, Wilco Tomassen^5^, Martijn Rooseboom^6^, Richard A. Currie^7^, Cyrille Krul^4^, Raymond Pieters^4,8^, Vera van Noort^1,9^^, and Marjolein Wildwater^3^^

**Content**

[Data sources 2](#_Toc115959018)

[Phenotype pathway enrichment analysis 4](#_Toc115959019)

[Pathway-to-phenotype mapping 9](#_Toc115959020)

[Tutorial 12](#_Toc115959021)

[Case study 32](#_Toc115959022)

[References 42](#_Toc115959023)

# Data sources

The DARTpaths application makes use of its own internal database, which can be filled with data from various sources. Some data sources do not allow redistribution and others may require a license for e.g., non-academic usage. Therefore, users are encouraged to create their own scripts for extraction, transformation and loading (ETL) of data. In that way they can ensure that the imported data fits their needs and licenses. Moreover, it allows them to add non-public data from in-house academic or enterprise sources or external commercial data vendors. For a number of public data sets (see Table 1), example ETL script are shared with the DARTpaths source code under the same Apache 2.0 license.

**Table S1**. Data sources and licenses.

| Data source | Data type | URL | Terms and conditions | ETL |
| --- | --- | --- | --- | --- |
| Concawe | Substance categories | [https://www.concawe.eu](https://www.concawe.eu/) | (not specified) | Downloaded by ETL scripts |
| Dictybase | Mutant phenoytpes | [http://dictybase.org](http://dictybase.org/) | (not specified) | Downloaded by ETL scripts |
| ECHA | Substances | <https://echa.europa.eu/> | <https://echa.europa.eu/nl/legal-notice> | Users needs to confirm terms and conditions before download |
| ECHA | Classification, Labelling and Packaging (CLP) regulation | <https://echa.europa.eu/> | <https://echa.europa.eu/nl/legal-notice> | Users needs to confirm terms and conditions before download |
| Ensembl | Orthology data (cross-species homologous genes) | [https://www.ensembl.org](https://www.ensembl.org/) | <https://www.ebi.ac.uk/about/terms-of-use/> | Downloaded by ETL scripts |
| EPA | Substances | [https://comptox.epa.gov](https://comptox.epa.gov/) | <https://comptox.epa.gov/dashboard/about> | Downloaded by ETL scripts |
| EPA | In vitro cell assays | [https://comptox.epa.gov](https://comptox.epa.gov/) | <https://comptox.epa.gov/dashboard/about> | Downloaded by ETL scripts |
| FlyBase | Genotype-phenotype associations | <https://flybase.org/> | <https://wiki.flybase.org/wiki/FlyBase:About#FlyBase_Copyright> | Downloaded by ETL scripts |
| International Mouse Phenotyping Consortium | Genotype-phenotype associations | [https://www.mousephenotype.org](https://www.mousephenotype.org/) | <https://www.mousephenotype.org/about-impc/terms-of-use> | Downloaded by ETL scripts |
| LOA-Reach consortium | Substance categories | <https://loa-reach.com/> | (not specified) | Shared in dartpaths repository |
| MGI | Genotype-phenotype associations | <http://www.informatics.jax.org/> | <http://www.informatics.jax.org/mgihome/other/copyright.shtml> | Downloaded by ETL scripts |
| OBO | Ontologies | [http://obofoundry.org](http://obofoundry.org/) | [http://obofoundry.org lists licenses for individual ontologies](http://obofoundry.org/) | Downloaded by ETL scripts |
| QSARtoolbox | In vivo substance activity (regulatory studies) | [https://qsartoolbox.org](https://qsartoolbox.org/) | http://www.oecd.org/termsandconditions , https://echa.europa.eu/nl/legal-notice | Users need to register before download |
| Reactome | Biological pathways | <https://reactome.org/> | <https://reactome.org/license> | Downloaded by ETL scripts |
| Reactome | Mapping from Ensembl genes to Reactome pathways | <https://reactome.org/> | <https://reactome.org/license> | Downloaded by ETL scripts |
| Wormbase | Phenotypes observed after exposure to substance | [https://wormbase.org](https://wormbase.org/) | <https://wormbase.org/about/policies> | Downloaded by ETL scripts |
| ZFIN | Substances | [https://wiki.zfin.org](https://wiki.zfin.org/) | <https://wiki.zfin.org/display/general/WARRANTY+AND+LIABILITY+DISCLAIMER%2C+OWNERSHIP%2C+AND+LIMITS+ON+USE> | Downloaded by ETL scripts |
| ZFIN | Phenotypes observed after exposure to substance | [https://wiki.zfin.org](https://wiki.zfin.org/) | <https://wiki.zfin.org/display/general/WARRANTY+AND+LIABILITY+DISCLAIMER%2C+OWNERSHIP%2C+AND+LIMITS+ON+USE> | Downloaded by ETL scripts |
| (Dartpaths consortium) | Mammalian phenotypes extracted from literature (demo) |  | Apache 2.0 license | Shared in dartpaths repository |
| (Dartpaths consortium) | Fine-grained orthology additions (demo) |  | Apache 2.0 license | Shared in dartpaths repository |

# Phenotype pathway enrichment analysis

| **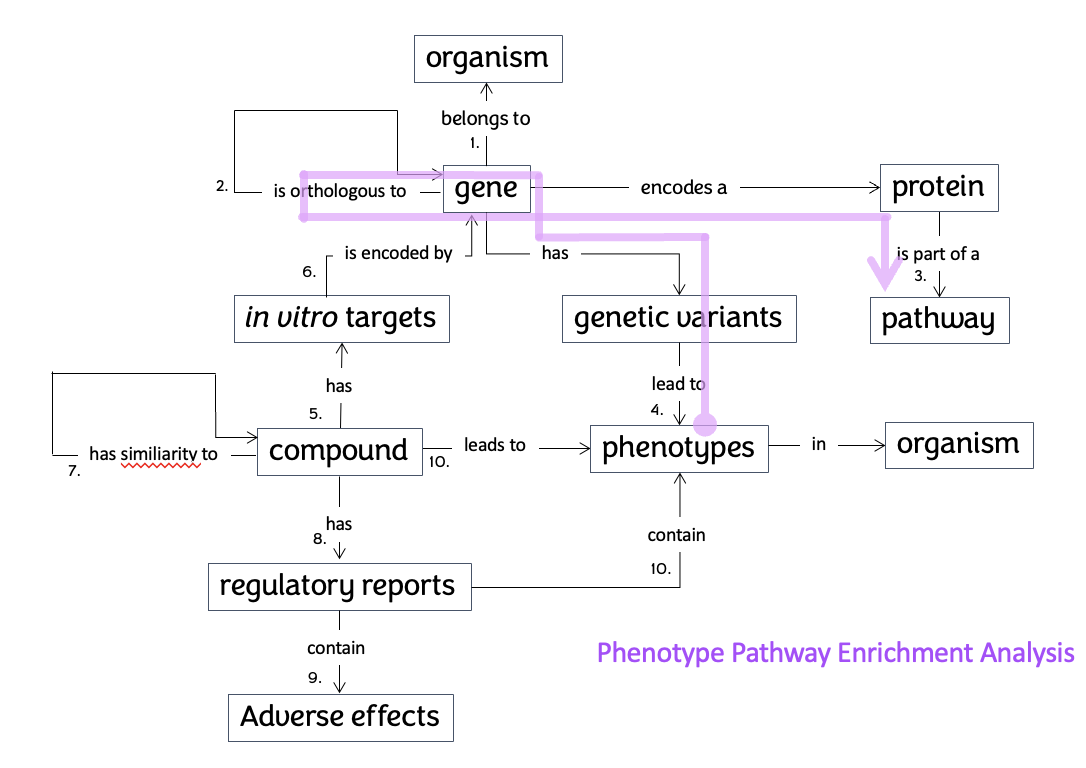** |
| --- |
| \| **Figure S1.** The Phenotype Pathway Enrichment Analysis aspect (shown in purple) within the semantic data model of Xpaths. \| \| --- \| |

**Specificity of phenotypes and genes**

The specificity of phenotypes is calculated using the following steps:

The information content (IC) score is calculated for each phenotype based on the number of gene associations through genetic variants (see equation 1). Here, *f* represents the number of genes that the current phenotype is associated with, whereas *n* represents the total number of associations between all phenotypes and all genes (i.e., a constant). (For example, an aspecific *C. elegans* phenotype such as “embryonic lethal” that occurs 3,249 times has an IC of 4.6, while a more specific phenotype such as “anterior ganglion development variant” that occurs only once has an IC of 16.3.)

$IC=-\log_{2} \left( \frac{f}{n} \right)$ (1)

A weighted IC is calculated so that it ranges from 0 (i.e., least specific) to 1 (i.e., most specific) and thereby normalized between species (see equation 2). Thus, the specific IC value that would become a 0 or 1 depends on the species-specific distribution of IC values.

$ICweight=\frac{IC-min\left( IC \right)}{\max\left( IC \right)-min(IC)}$ (2)

The specificity of each gene is calculated as the average of the weighted ICs of phenotypes associated with that gene.

**Pathway prediction from phenotypic endpoints**

The pathway prediction module uses an area under the curve (AUC) algorithm to find the best matching pathways (see Figure S2) for a set of phenotypes induced by a compound. It does this by using the following steps:

Each *phenotype* is represented by a vector of all genes that are either associated or not (1 = hit and 0 = no hit).

A *profile* vector is created of all genes that are associated with at least one phenotype.

The *pathways* that belong to the genes in the profile vector are looked up, and the pathway genes are marked (1 = associated, 0 = not associated), creating a pathway-specific *query*.

The *profile* vector with genes each has a *specificity* value (i.e., average of weighted information score of all associated phenotype for that gene). Only phenotypes with a weighted IC of 0.2 and higher are used.

The *profile* vector is sorted in descending order (i.e., ranked) based on the *specificity* of genes.

The pathway-specific *query* vector is sorted according to the *profile* vector. In case of ties, hits are ranked higher than non-hits.

A *curve* is drawn based on a cumulative sum of the *query* vector (i.e., each element is the value of the corresponding *query* vector element plus the sum of all preceding elements in the same vector).

To get the *AUC* value, this new *curve* vector is summed. The maximum possible *AUC* value for each pathway is determined by the number of hit genes in the *query*, assuming all hit genes are ranked highest in the *query*.

| 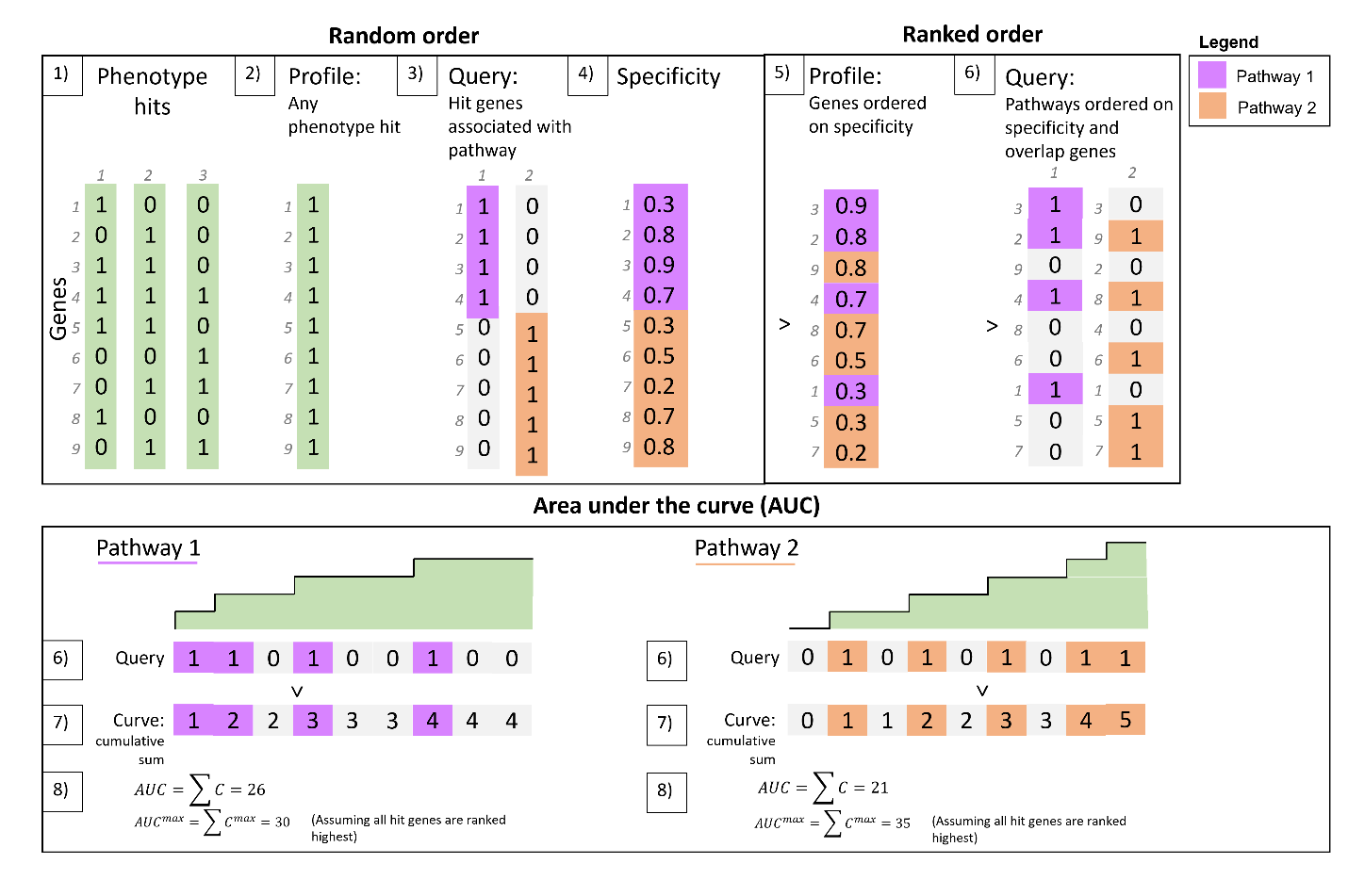 |
| --- |
| **Figure S2.** Example of pathway prediction.  *Note.* Numbers 1-8 correspond to the steps explained in the ‘Pathway prediction from phenotypic endpoints’ section. |

**Pathway *p*-value calculation**

*p*-values of predicted pathways are calculated by using Monte Carlo simulations (see Figure S3). It uses this to calculate the chance of false positive pathways, using the following steps:

Do 1000 simulations to randomly draw the same number of phenotypes as was used as input for the pathway prediction, and for the same species. (For example, say you have calculated the AUC value based on three phenotypes, then a simulation will be done a 1000 times by drawing a random set of three phenotypes and calculating the AUC value for each predicted pathway, given those three random phenotypes.)

The AUC value of each pathway, compared to the maximum possible AUC value for that pathway (given its size and query order), will be calculated as a percentage. This is done both for the true set of phenotypes as well as for the random set of phenotypes for each simulation.

Calculate the number of false positive pathways by counting the number of simulations where the random AUC percentage was larger than the actual AUC percentage, i.e. the pathway was more likely to be affected at random.

For each species, calculate the *p*-value as the chance of false positives, by using a ratio between the percentage of false positive pathways over the number of simulations (equation 4). For example, say that after 1000 simulations, 50 simulations showed a larger hit percentage than the actual hit percentage, i.e., there are 50 positives, then the *p*-value becomes 0.051 (i.e., 50+1/1000+1).

Calculate the harmonic mean of cross-species *p*-values, combining the *p*-values for mammalian, *C. elegans*, and zebrafish phenotypes (equation 5) (Wilson, 2020).

The *p*-values for all pathways are then corrected for multiple comparisons using the false discovery rate (Benjamini & Hochberg, 1995; equation 8). FDR-corrected *p*-values < .05 are considered significant.

$p=\frac{\mathrm{Number}of false \mathrm{positive}s+1}{\mathrm{Total}\mathrm{number}\mathrm{of}simulations+1}$ (4)

$p_{harmonic mean}=\frac{1}{mean(\frac{1}{p_{mammalian},p_{C.elegans},p_{zebrafish}})}$ (5)

| 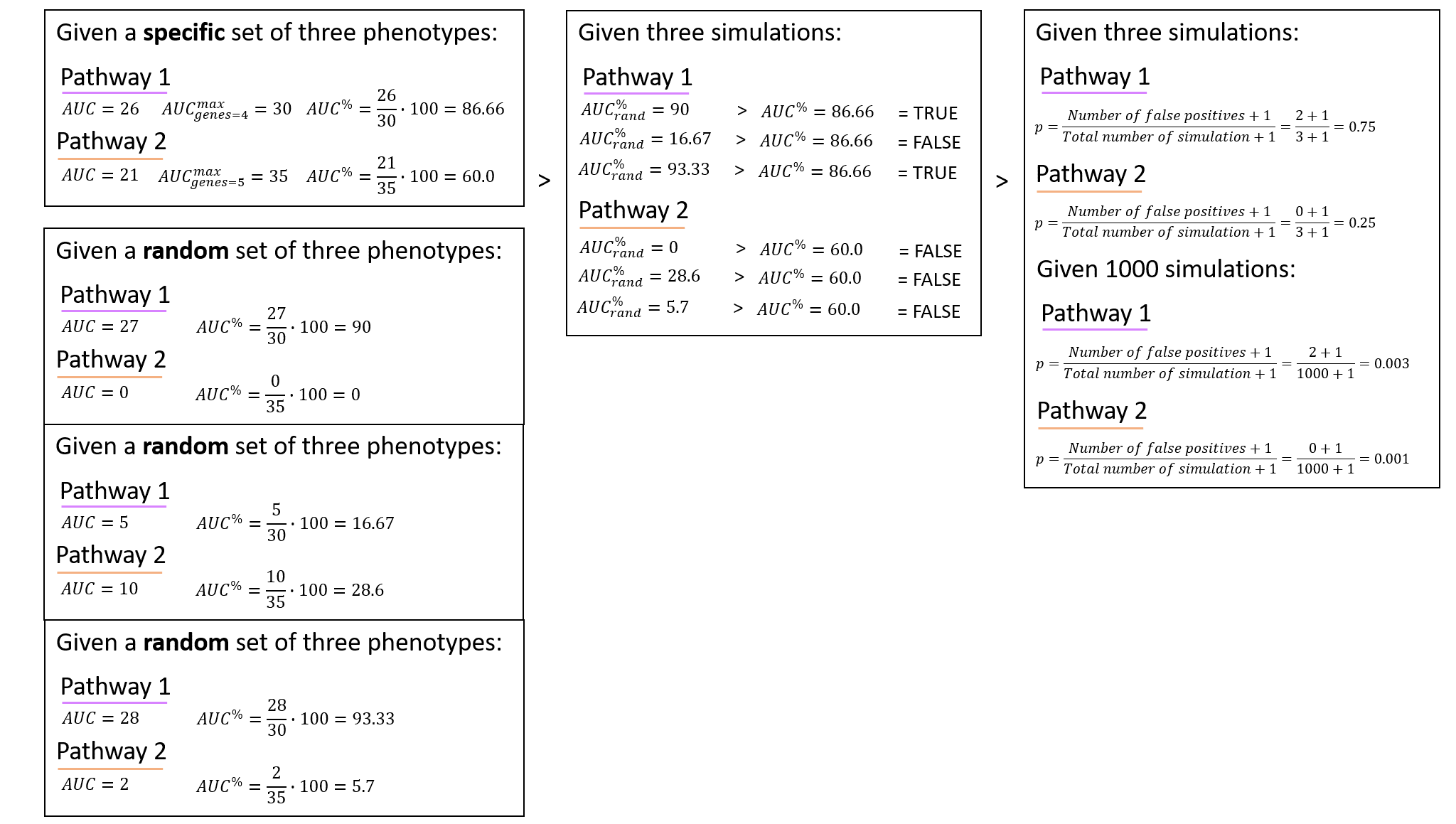 |
| --- |
| **Figure S3.** Example of *p*-value calculation for pathways. |

**Summary at Reactome hierarchy level**

The DARTpaths application can be configured to summarize the pathway prediction by showing only Reactome pathways at a certain hierarchical level (typically level 3 and 4). In that case, the displayed *p*-value is the minimum of the *p*-values of that pathway and its sub-pathways.

In addition to the phenotype data, our database also contains in vitro assay data. Typically, the activity of a compound (or substance) is measured in a number of gene assays. If the potency of the compound for an assay is higher than a pre-defined threshold (e.g. pAC50 value), this is considered a hit. We use a Jaccard index to calculate an “in vitro score” for each pathway (equation 6).

$IJ\left( A,B \right)=\left| A \cap B \right|/|A \cup B|$ (6)

*A* represents the set of *in vitro* targets targeted by the compound and *B* is the set of *in vitro* targets present on the pathway for which an in *in vitro* assay has been carried out. This score describes the overlap between the hit genes for the compound and the genes that were measured for that compound (hit or no hit) that are part of the pathway of interest. This score is only calculated for human Reactome pathways. Non-human genes are mapped to their human orthologs before calculating the score. If the same gene is measured in multiple assays or experiments, it is counted only once and considered a hit if there is at least one hit for the gene for the compounds of interest.

$In vitro score=\frac{n hit genes in pathway}{\left( n hit genes+n pathway genes measured \right)-n hit genes in pathway}$ (7)

We do not combine the in vitro score (equation 7) with the *p*-values obtained from the phenotype pathway enrichment analysis to create a single score. This has various reasons. Firstly, as our current in vitro data does not originate from a unbiased genome-wide analysis, it is relatively sparse (only a small number of genes is measured for each pathway) and may be biased to certain genes or pathways. Secondly, the definition of a “hit” may be arbitrary in some cases. Thirdly, there may not be any relation between the number of hits in a pathway and the expected disruption of the pathway as the pathway architecture and kinetics are not captured in the current data and analysis. For example disruption of a receptor may have more impact than disruption of multiple downstream pathway components. Although it is not included in the primary ranking, the in vitro score can be useful as a secondary measure for ranking pathway relevance and is used as such in the DARTpaths application.

# Pathway-to-phenotype mapping

| **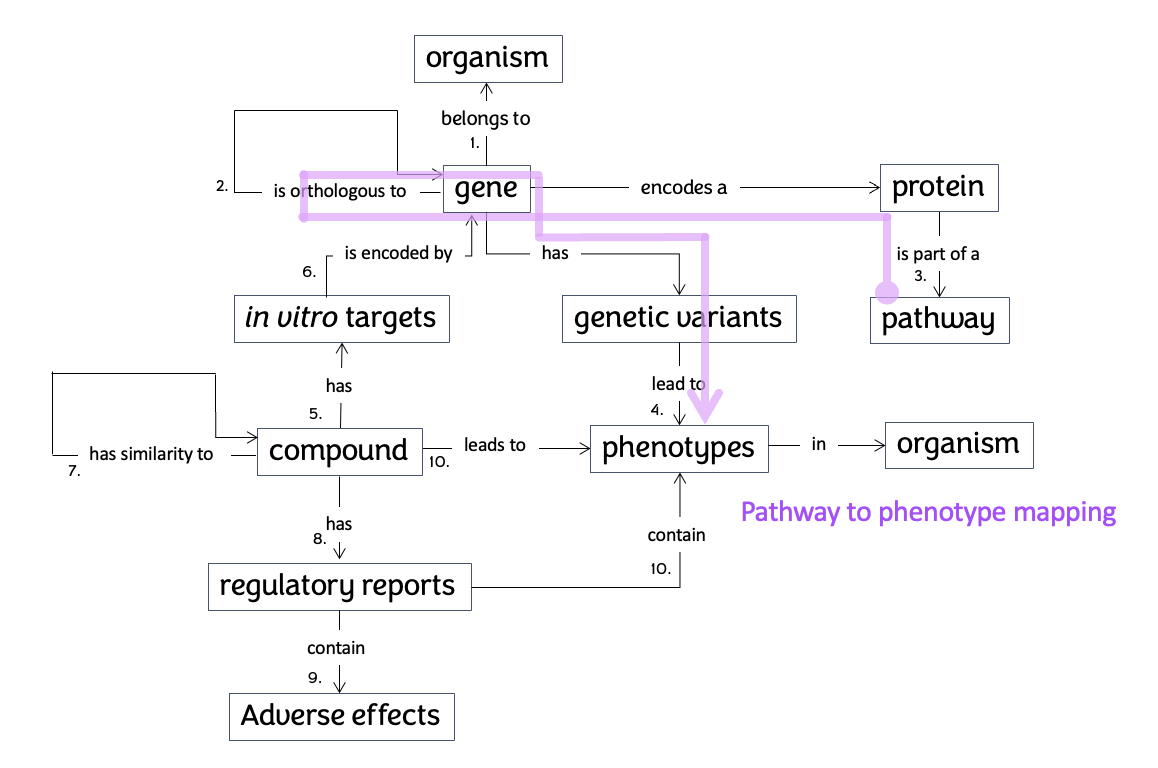** |
| --- |
| **Figure S4.** The Pathway to Phenotype Mapping aspect (shown in purple) within the semantic data model of Xpaths. |

**Phenotype prediction from genes**

The predicted phenotypic effects are found by mapping pathways to genes, mapping those to orthologous genes and finding enrichment of phenotypes among genetic variants of those orthologous genes. Mutant phenotypes characterize the consequence of disturbing or disrupting a gene’s information output. Genetic variation data with its corresponding effect on phenotypes is used to predict the effect of disturbing a pathway.

**Phenotype *p*-value calculation**

*p*-values are calculated based on hypergeometric distribution (equation 8).

$p=\frac{n!m!N!\left( m+n-N \right)!}{i!\left( n-i \right)!\left( N-i \right)!\left( m+i-N \right)!\left( m+n \right)!}$ (8)

where,

*n =* number of annotated genes with phenotype *x*

*m =* number of genes in the pathway that have phenotype annotation

*N =* total number of genes for that species that have phenotype annotation

*i =* count (n ∩ m)

$q=\frac{i}{M}\cdot Q$ (9)

**Adjusted *p*-values**

*P*-values are adjusted to control the False Discovery Rate (Benjamini & Hochberg, 1995) If the *p*-values are sorted as , the adjusted *p*-value corresponding to $p_{\left( i \right)}$ is given by equation 10 (Benjamini, Heller, Yekutieli, 2009).

$p_{\left( i \right)}^{BH}=\min\left( \min_{j\geq i} \left( \frac{mp\left( j \right)}{j} \right), 1 \right)$ (10)

Only phenotypes with $p_{\left( i \right)}^{BH}\leq0.001$ are considered significant.

# Tutorial


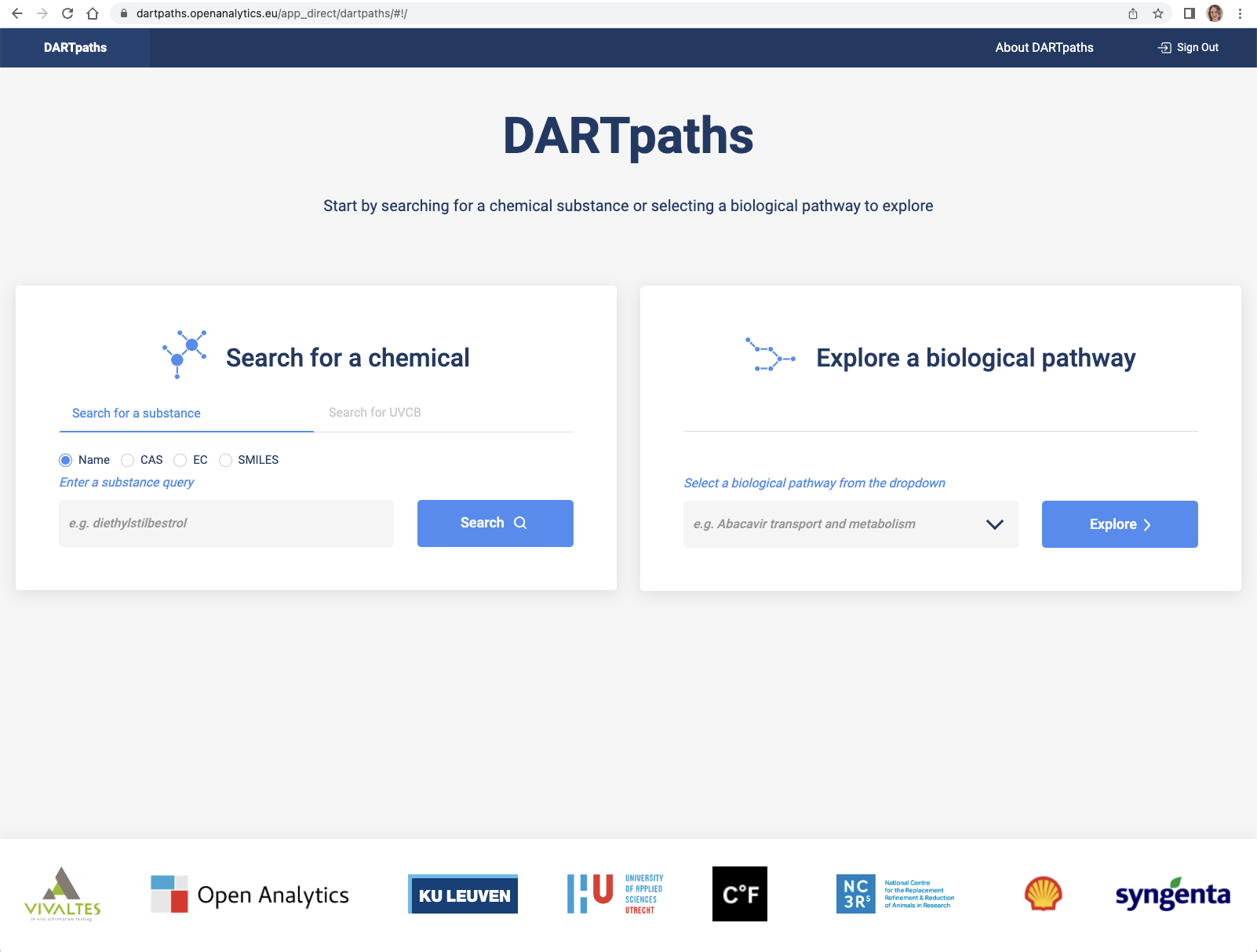


**Figure S5.** Landing page DARTpaths to search for a substance (left).

*Note.* You can search for a chemical by entering a name, CAS number, EC number or SMILES string.


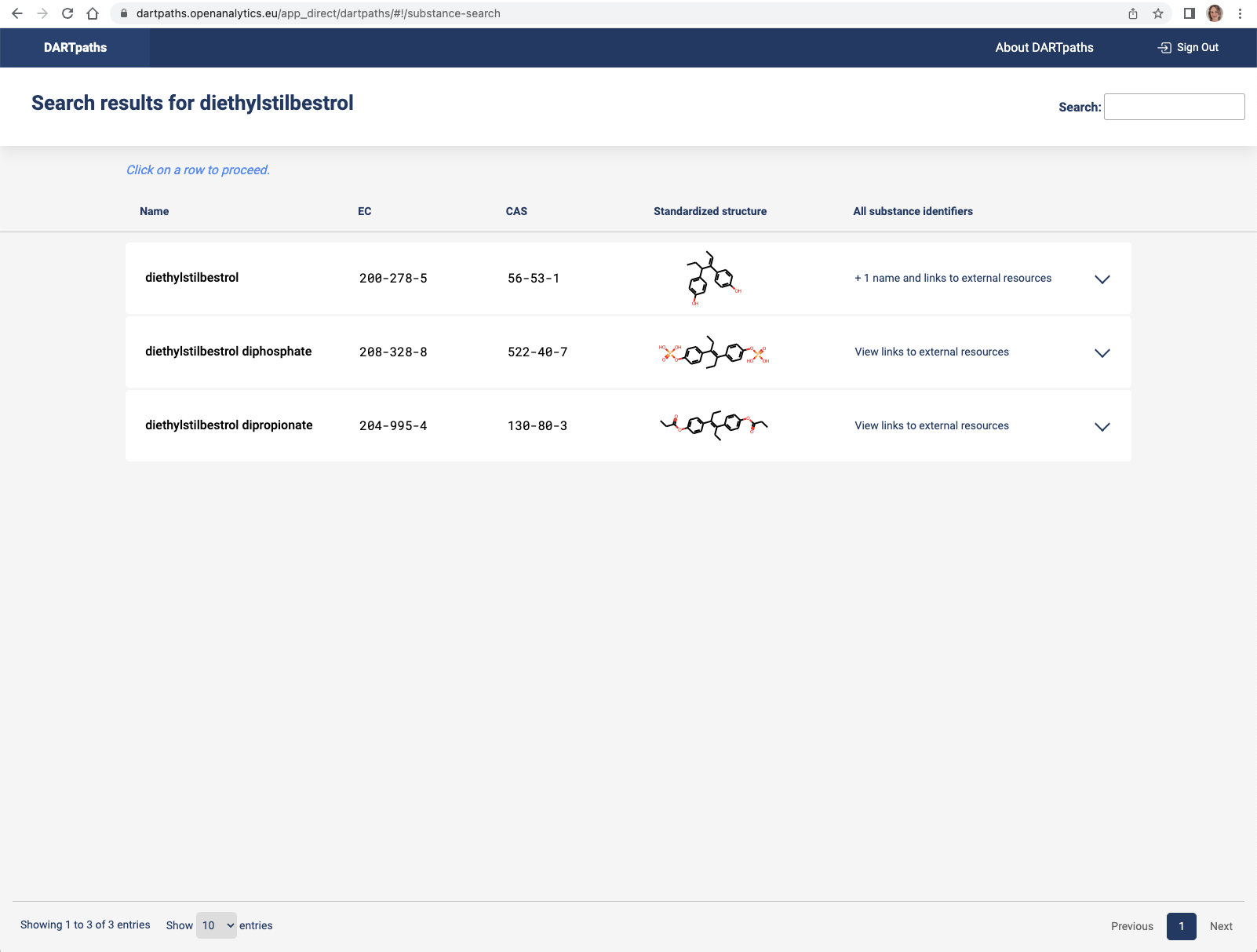


**Figure S6.** Search results for a substance.

*Note.* A list of matching substances is generated. By selecting one of the compounds, you will see detailed information for this compound.


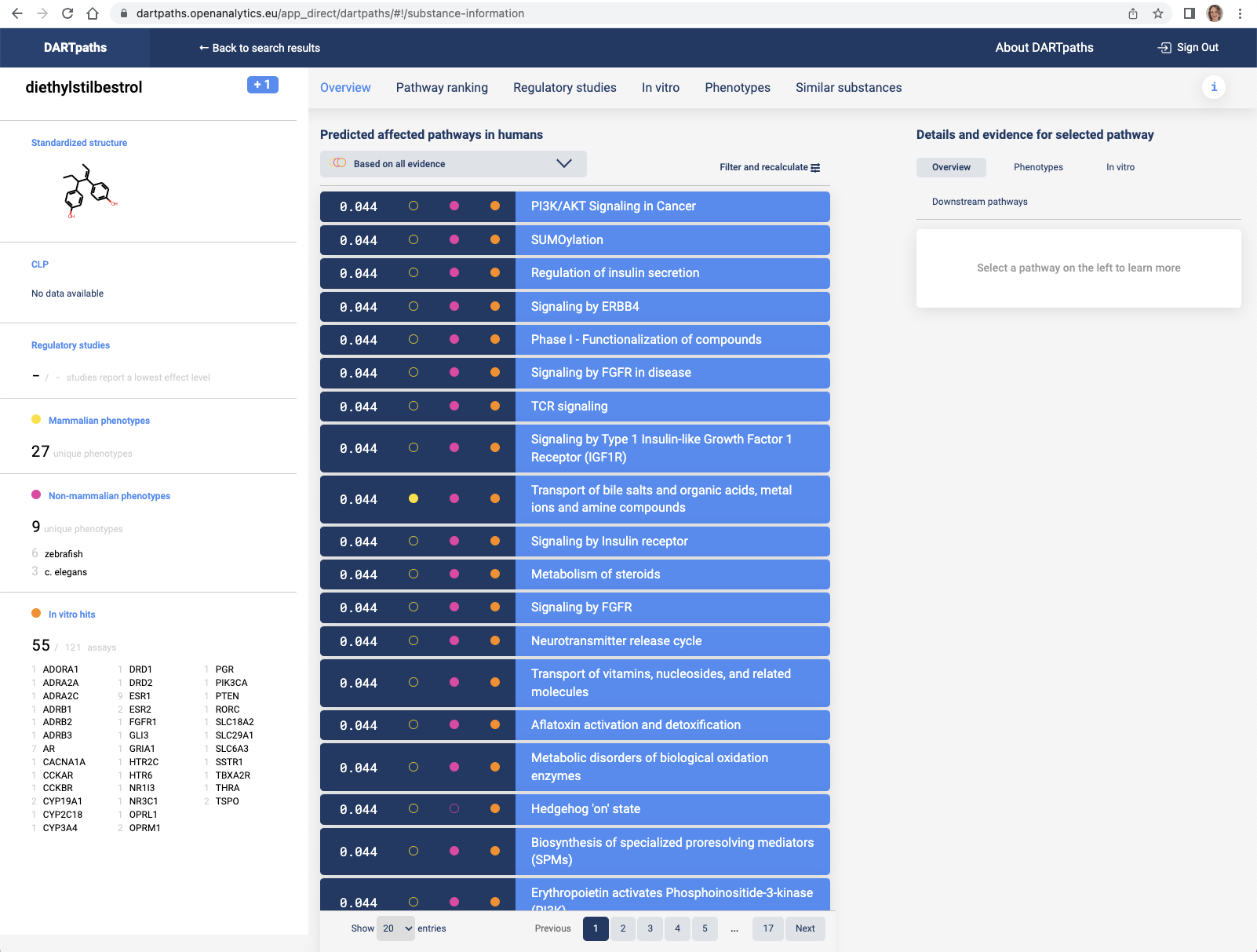


**Figure S7.** Main substance exploration page for diethylstilbestrol (DES).

*Note.* On the left, the name of the compound as well as the normalized structure is displayed. When regulatory studies are available, it is indicated how many of those studies report DART adverse effects with a lowest effect level. When phenotypes are present in the database, the number of phenotypes in different organisms is displayed and the total number of in vitro hits for this compound and the names of the targets.

In the middle the predicted affected molecular pathways are displayed, based on Mammalian phenotypes (yellow), Non-mammalian phenotypes (magenta) and in vitro assays (orange).

By selecting one of the pathways detailed information about the predicted pathway is presented on the right.


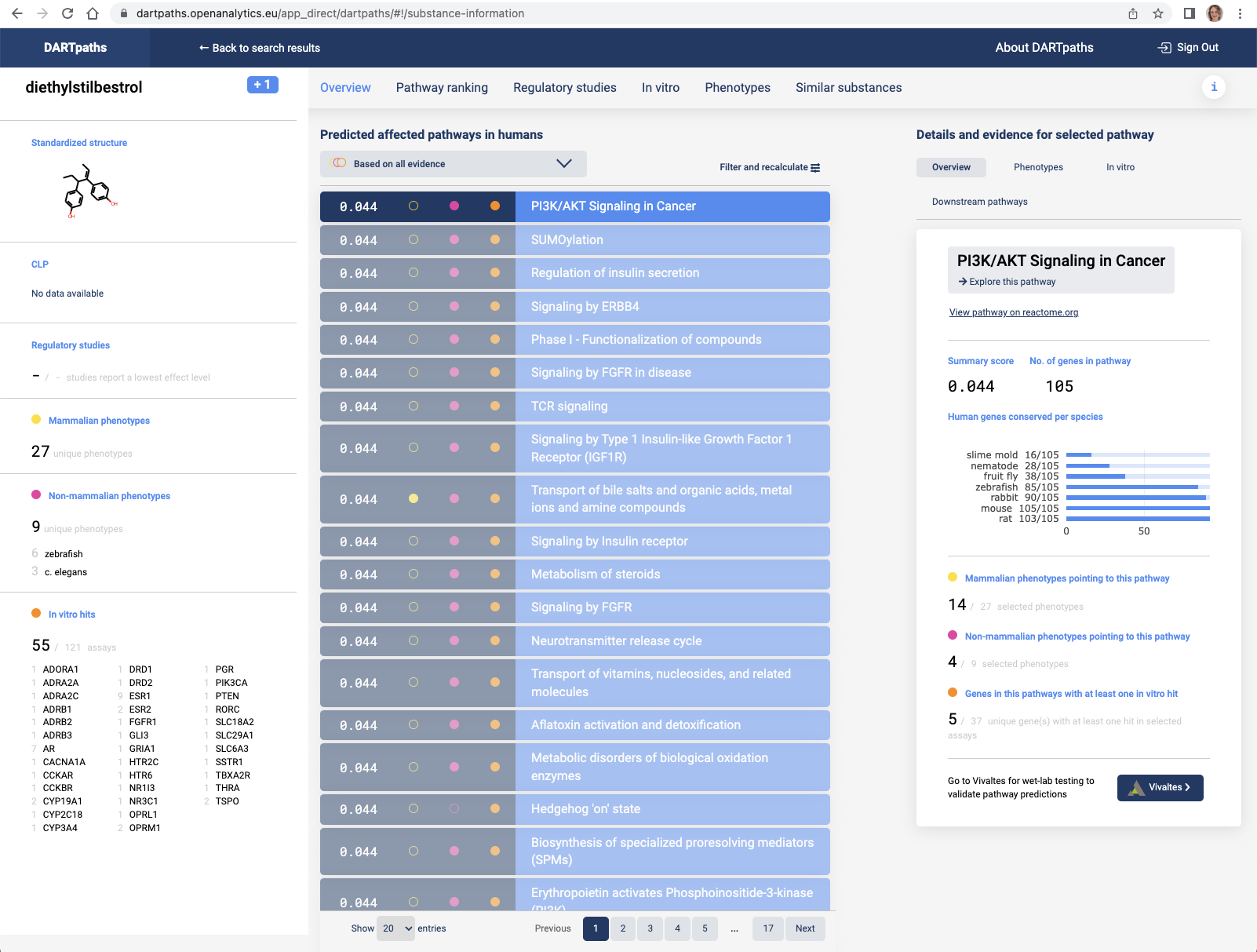


**Figure S8.** Overview of pathway selection in substance exploration.

*Note.* For the selected pathway, an overview of the conservation of the pathway in different model organisms is presented. This enables a user to assess if that model organism is useful to assess toxicity of this compound. The number of phenotypes that point to this pathway (via Phenotype Pathway Enrichment Analysis) is given (here 14 mammalian, 4 non-mammalian phenotypes).

In the right panel by clicking on Phenotypes the phenotypes that lead to the identification of this pathway are displayed.


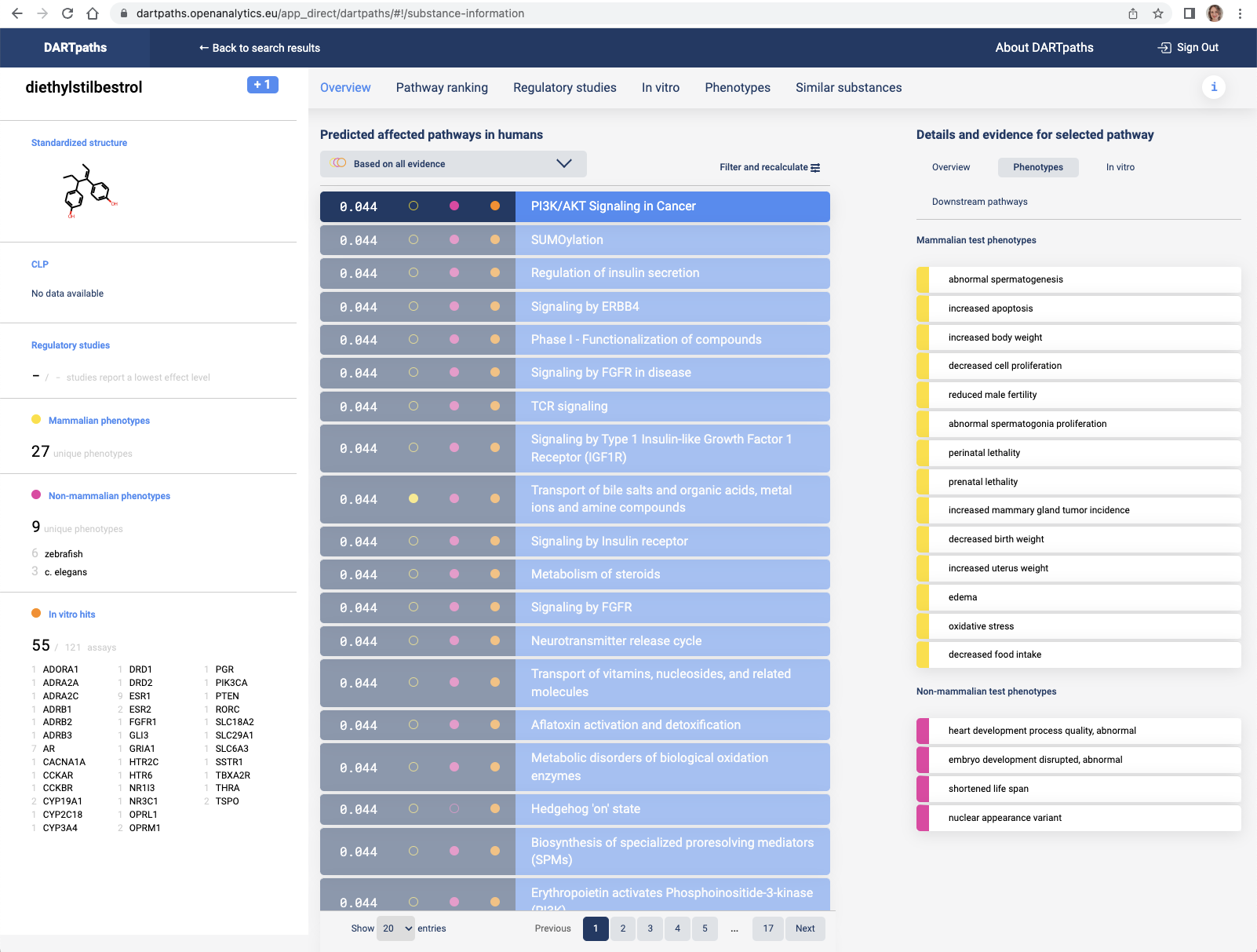


**Figure S9.** Phenotypes of pathway selection in substance exploration.

*Note.* By clicking on in vitro you see the in vitro targets that are present on the currently selected molecular pathway.

Using ‘filter and recalculate’, the phenotypes on which the Phenotype Pathway Enrichment Analysis is based on can be changed (eg based on expert knowledge which phenotypes are relevant).


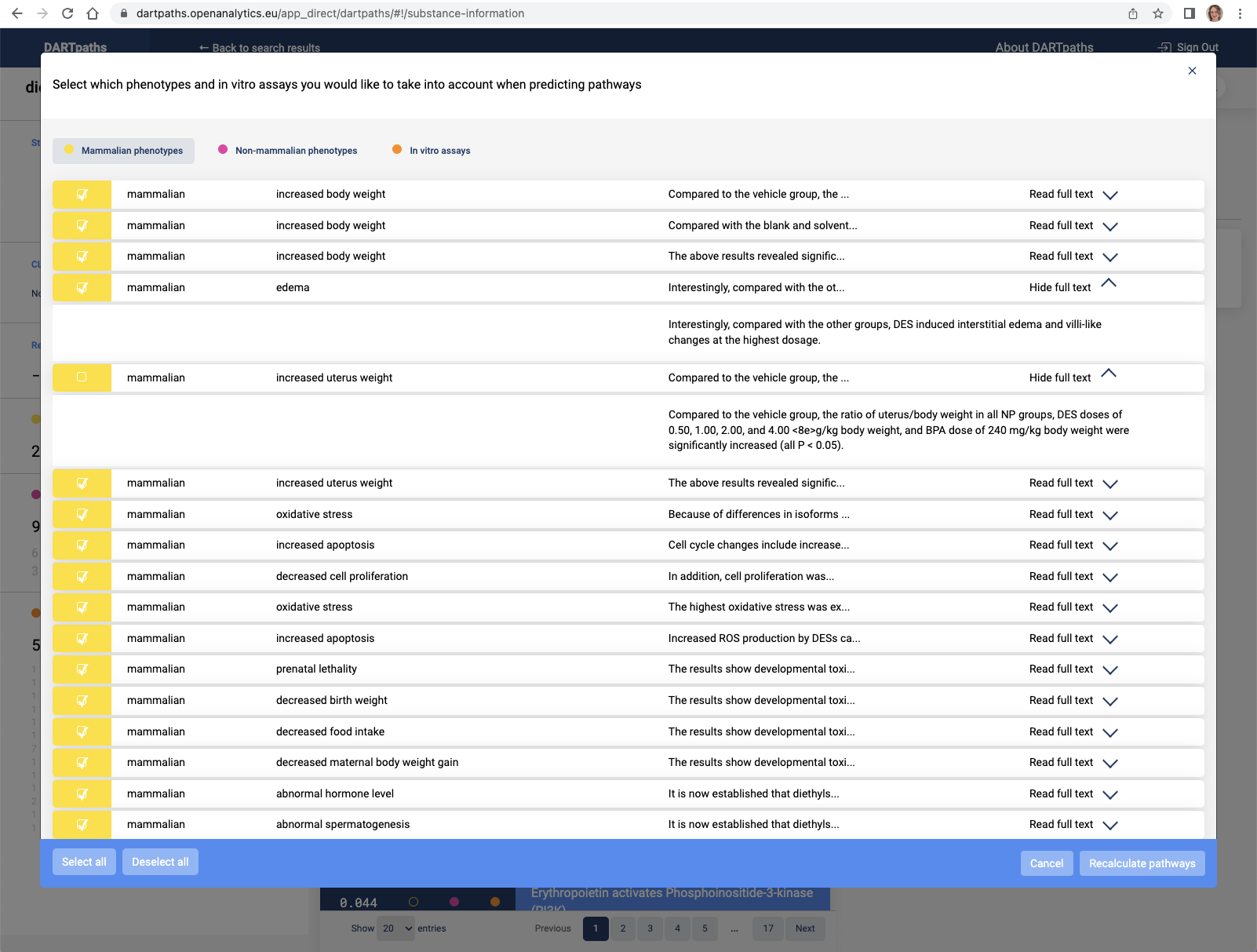


**Figure S10.** Filter and recalculate mammalian phenotypes in substance exploration.

*Note.* Clicking on ‘Read full text’ displays the text exerpt on which the Phenotype annotation is based.

In addition, the target information on which the prediction is based can be adapted


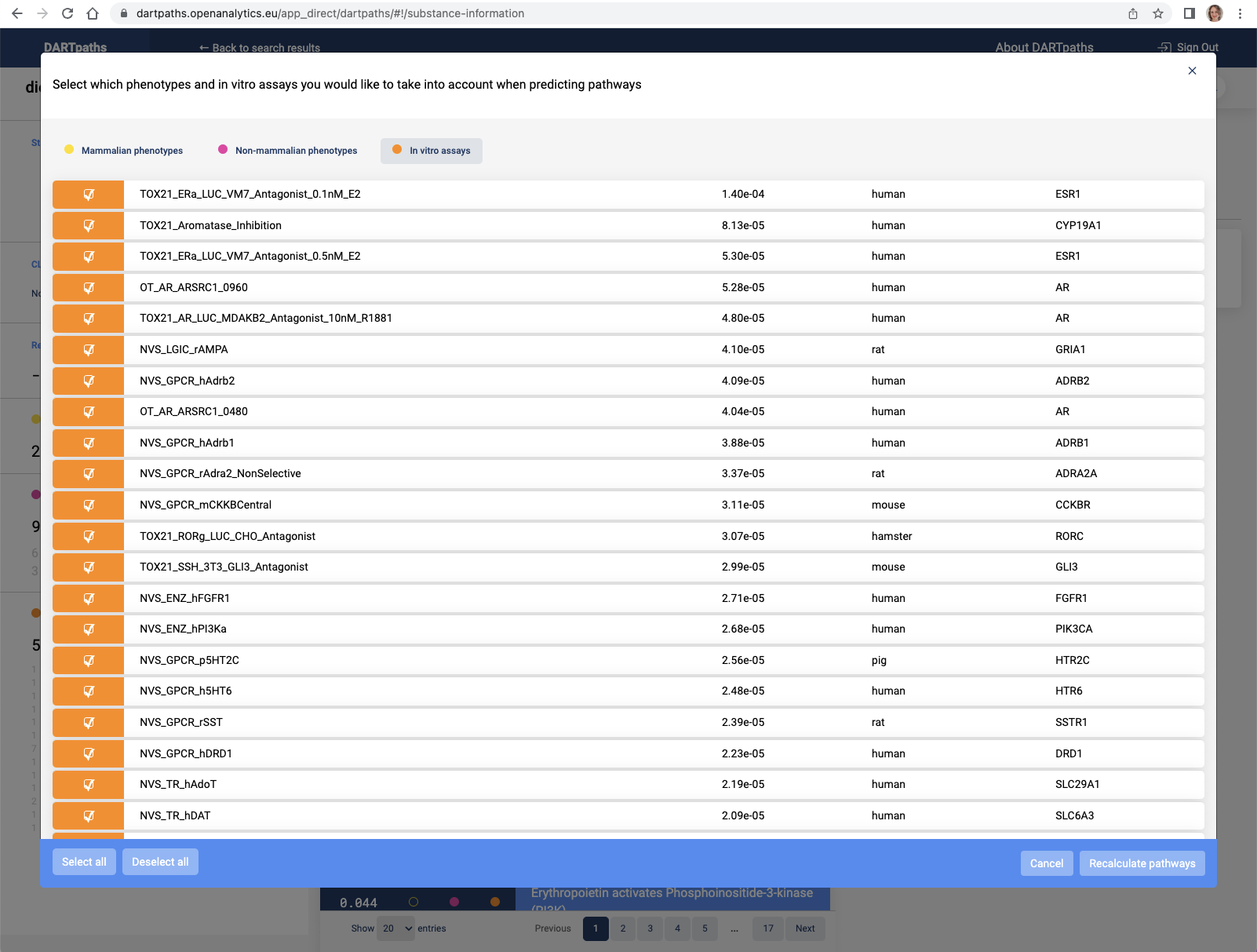


**Figure S11.** Filter and recalculate in vitro assays in substance exploration.

*Note.* Based on expert knowledge which in vitro targets are of relevance. A new ranking of pathways is then displayed:


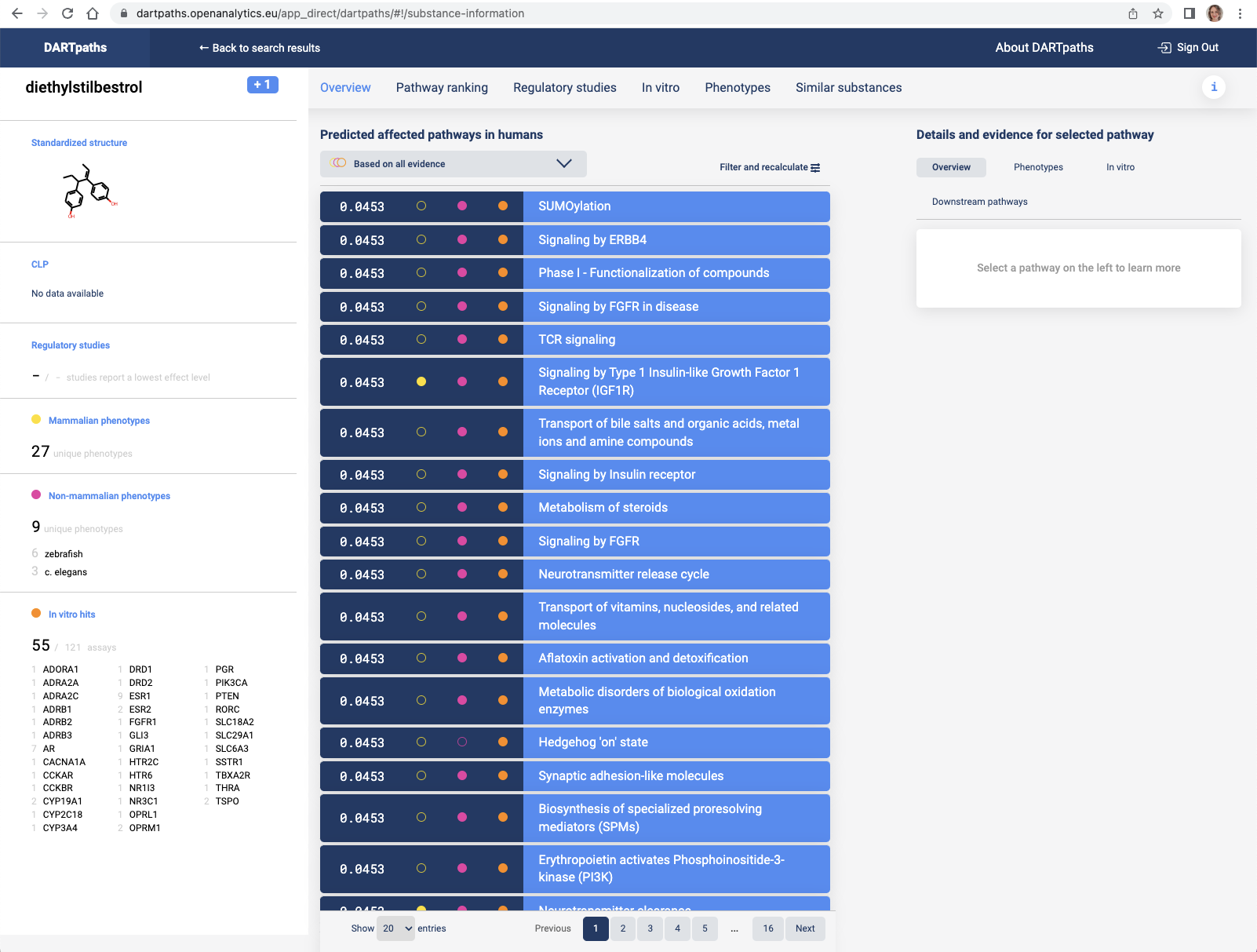


**Figure S12.** Main substance exploration page after using filter and recalculate.


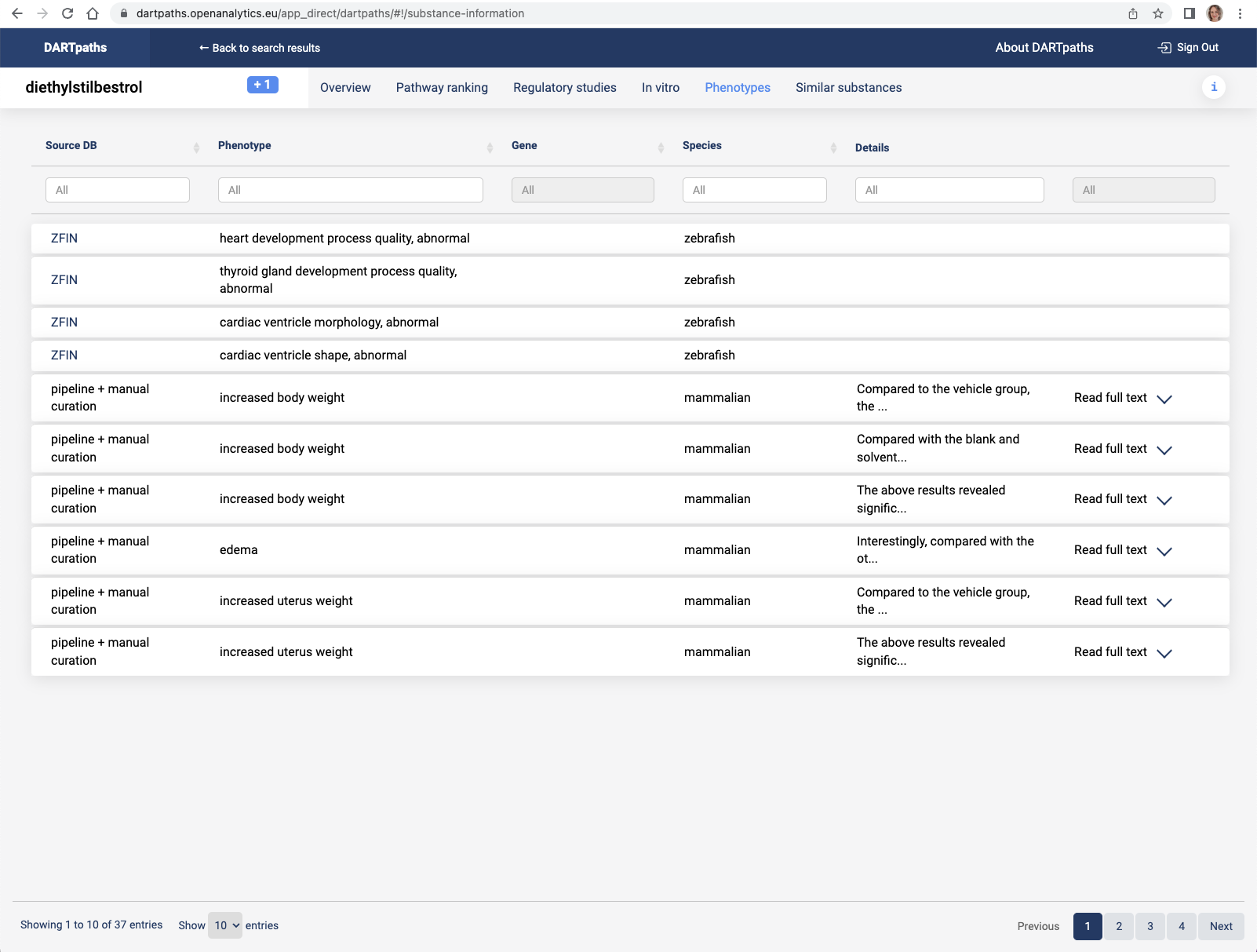


**Figure S13.** Phenotypes in substance exploration page.

*Note.* The links at the top (Pathway ranking, Regulatory studies, in vitro, Phenotypes, Similar substances) give detailed information. For example for phenotypes, the source of the phenotypic information is given.


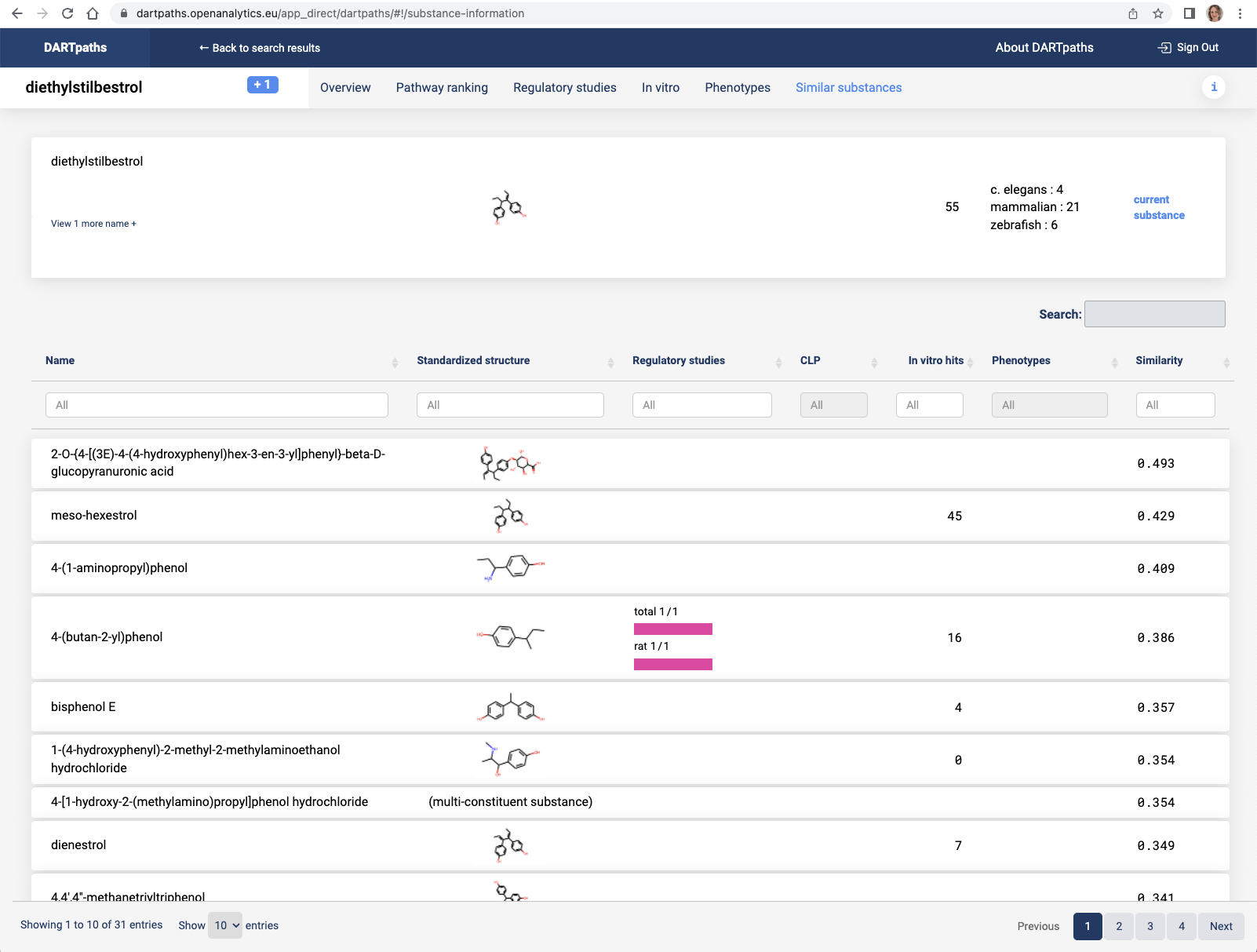


**Figure S14.** Similar substances in substance exploration page for diethylstilbestrol.

*Note. S*imilar substances leads to a page with compounds that are chemically similar to the currently selected compound.


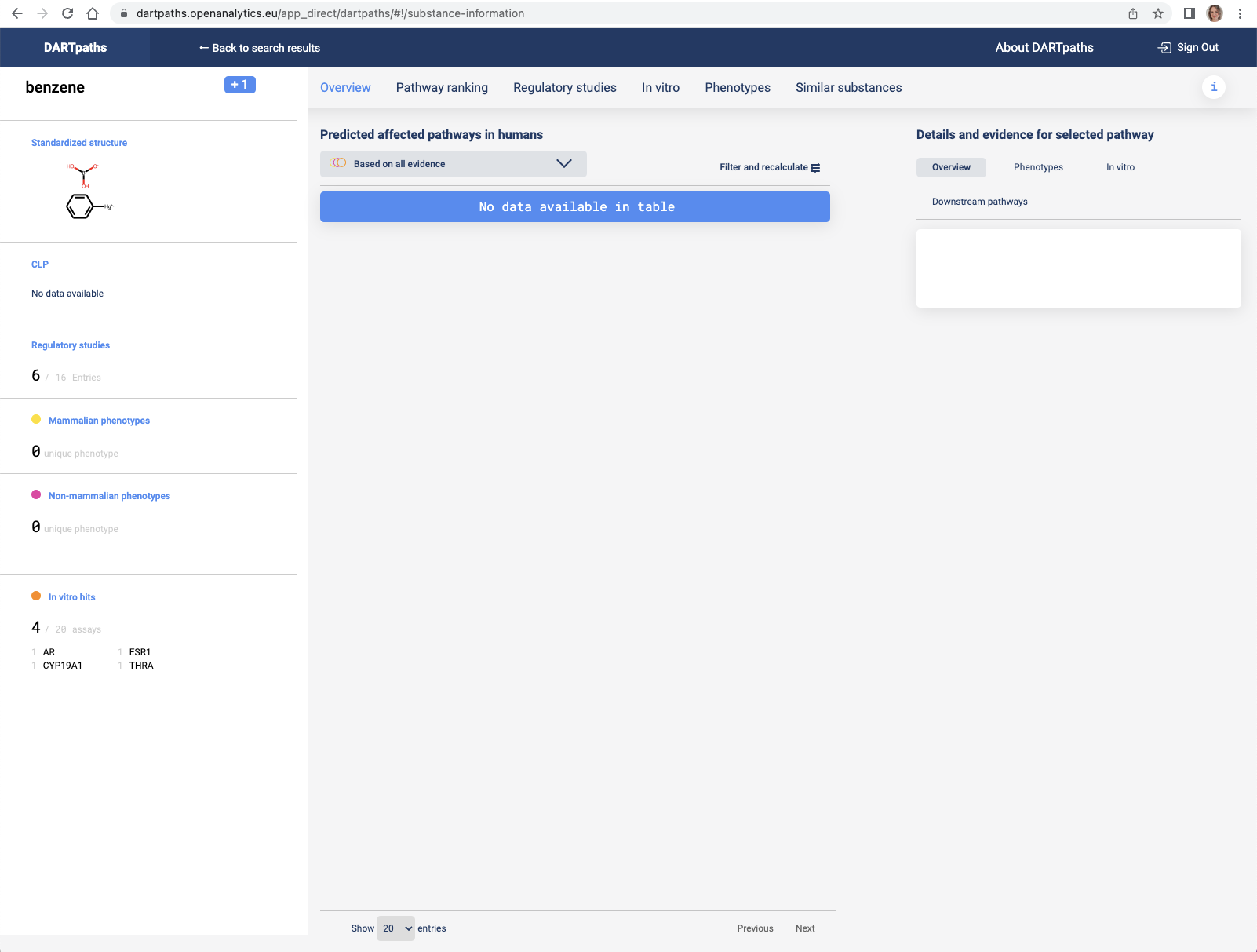


**Figure S15.** Main substance exploration page for benzene.

*Note.* Searching with benzene leads to a different overview.


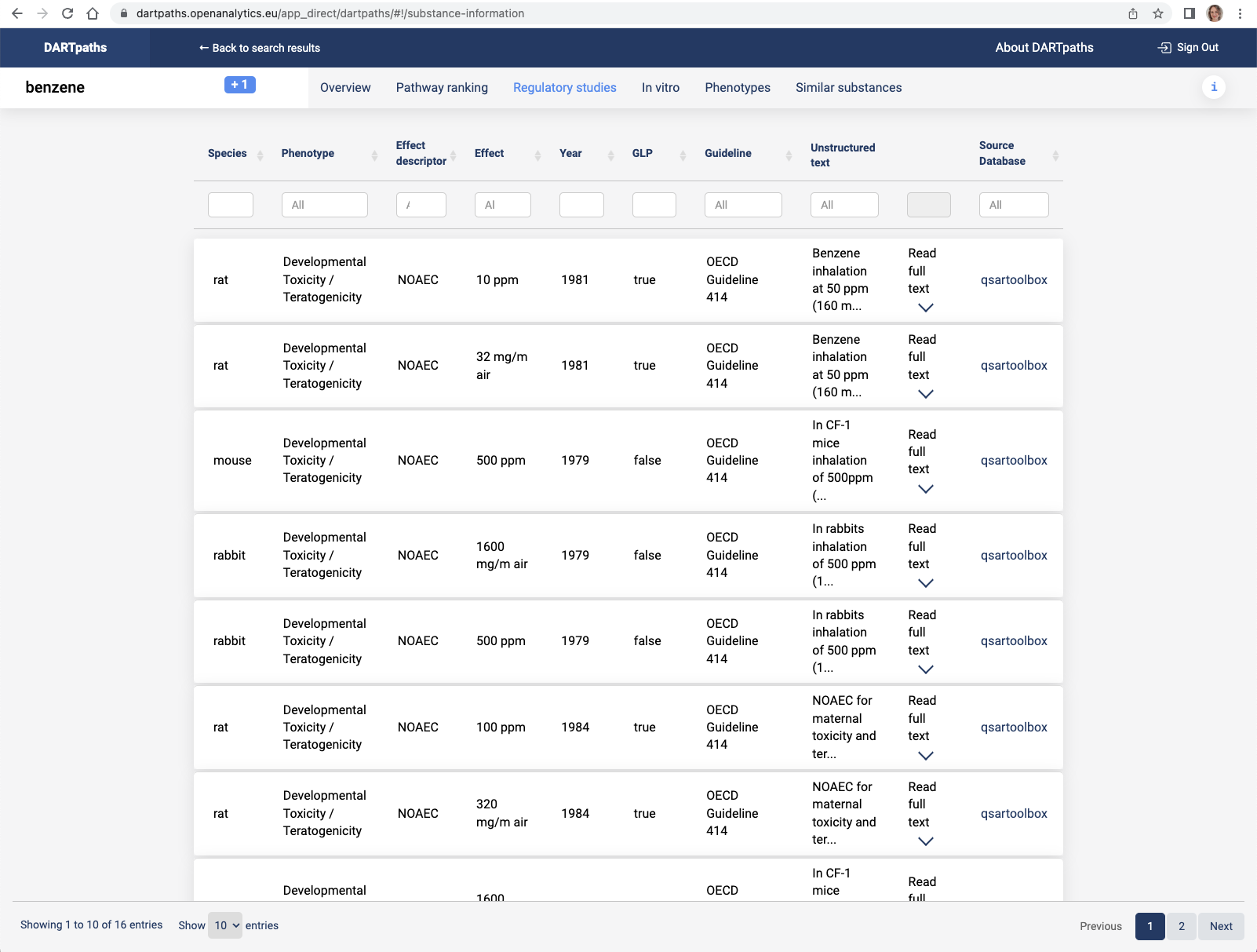


**Figure S16.** Regulatory studies in substance exploration page.

*Note.* For benzene, a large number of regulatory studies are available, for which an overview is provided.


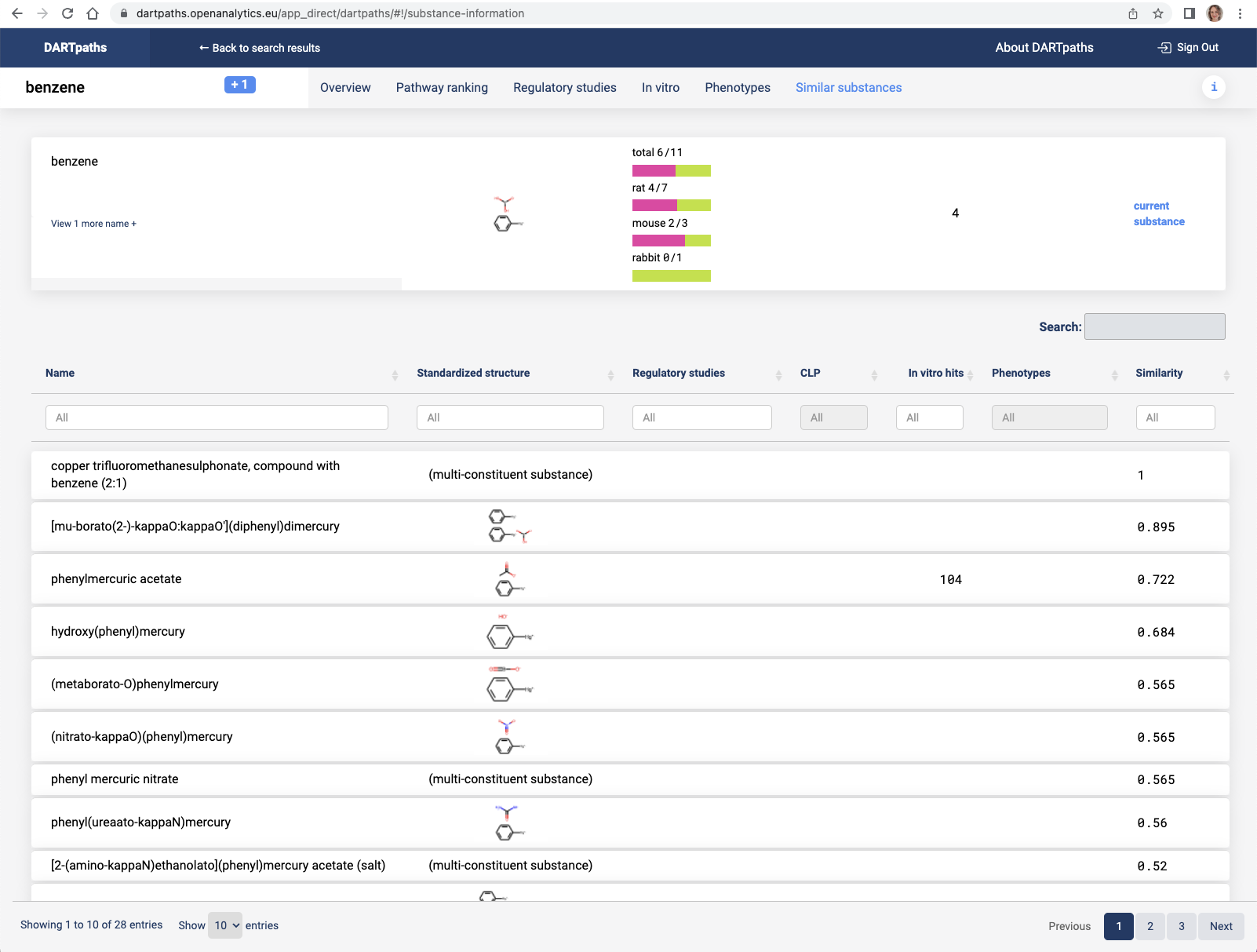


**Figure S17.** Similar substances in substance exploration page.

*Note.* In the tab similar substances an overview is give for how many of these regulatory studies find the compound to have adverse effects (magenta) for developmental and reproductive toxicity (DART)*.* And shows similar substances.


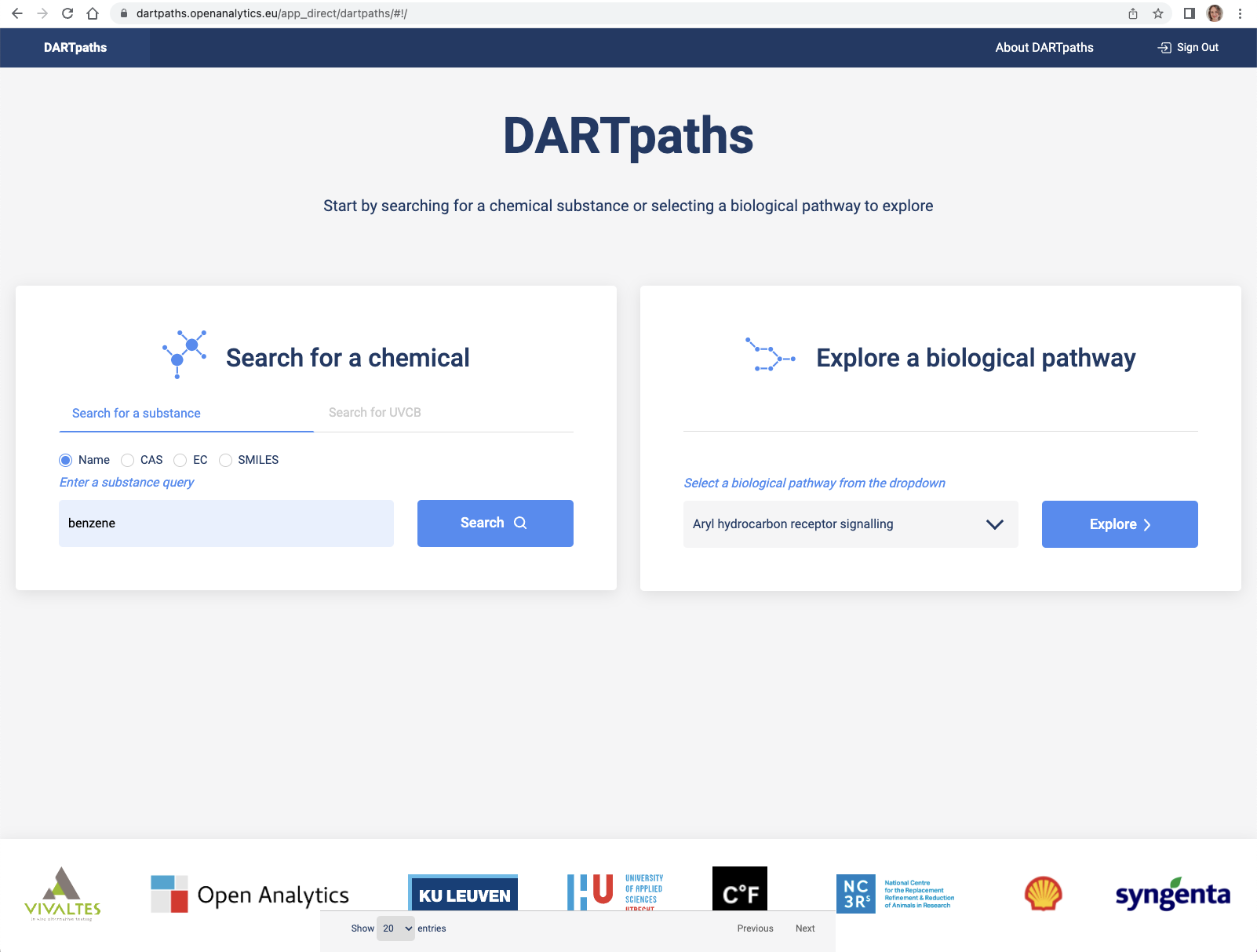


**Figure S18.** Landing page DARTpaths to explore pathways (right).

*Note.* If the molecular pathway is known, it is also possible to start from this end. Enter the name of a pathway or select from a list, here Aryl hydrocarbon receptor signalling is taken as an example.


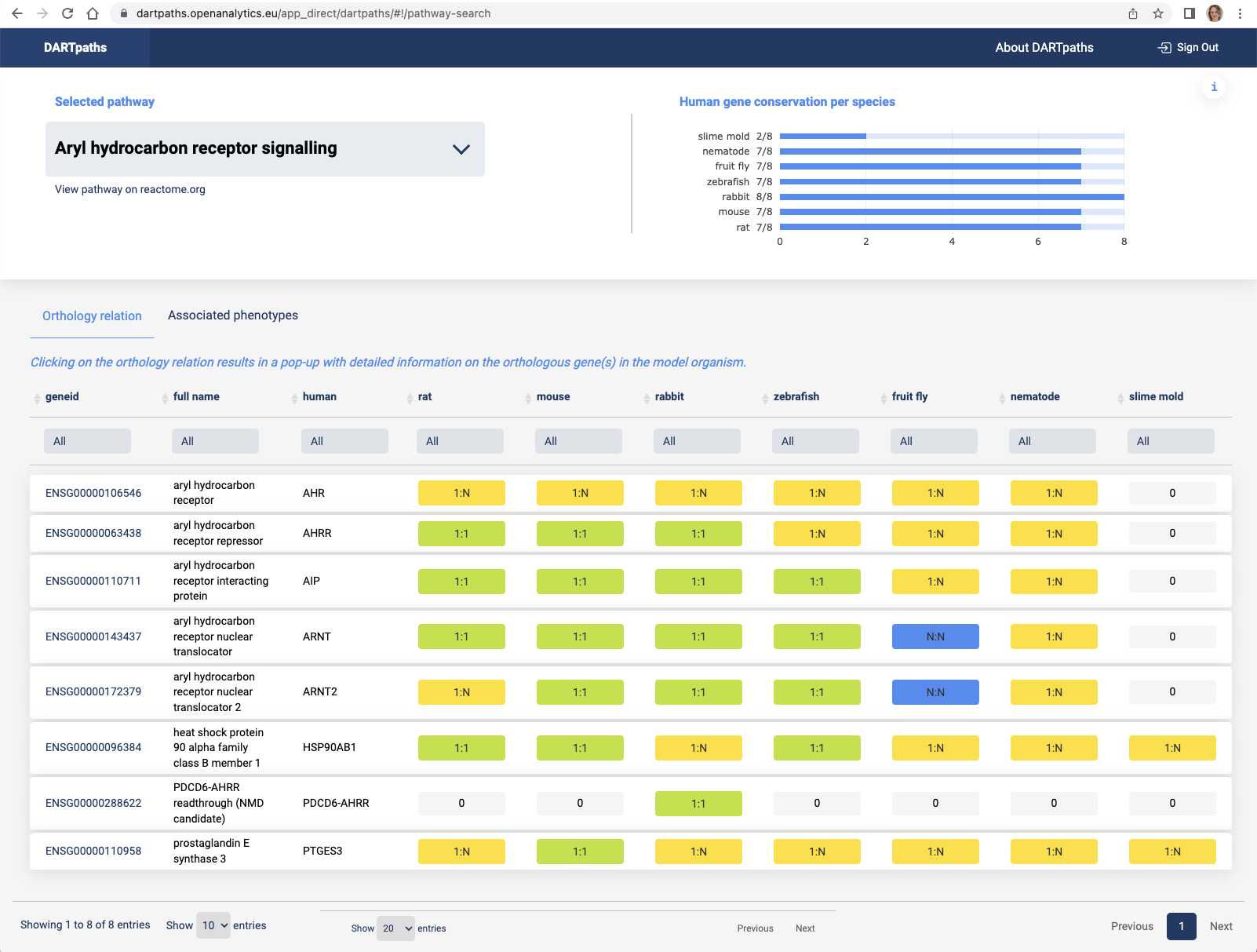


**Figure S19.** Orthology relation in main pathway exploration page.

*Note.* An overview of the conservation of all genes of the pathway in model organisms is provided at the top.

Details of which genes are present in each of the model organisms as 1-to-1 ortholog, 1-to-many ortholog or many-to-many ortholog.


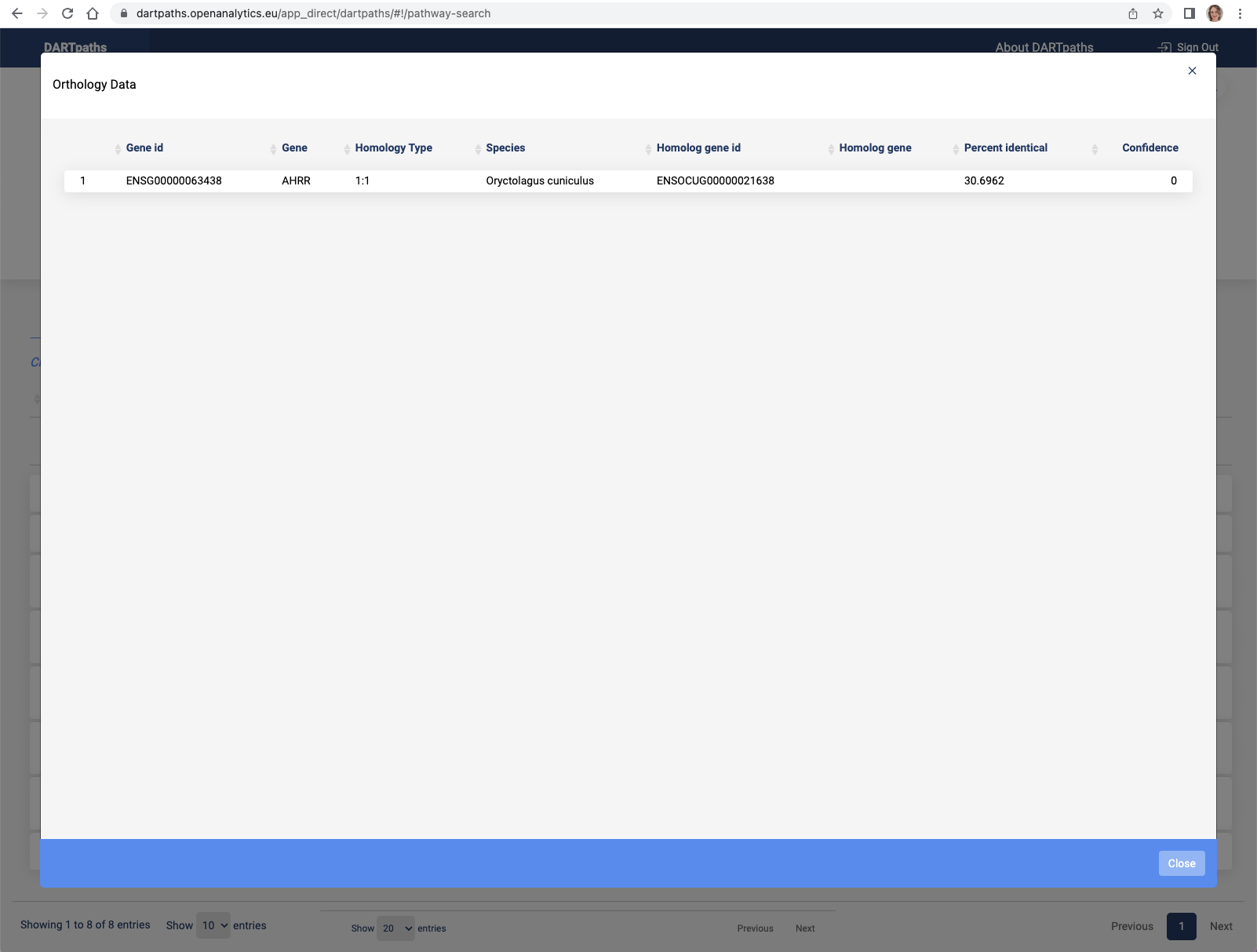


**Figure S20.** Orthology relation details in pathway exploration page.

*Note.* Clicking on the orthology relation gives the identity of the gene(s).


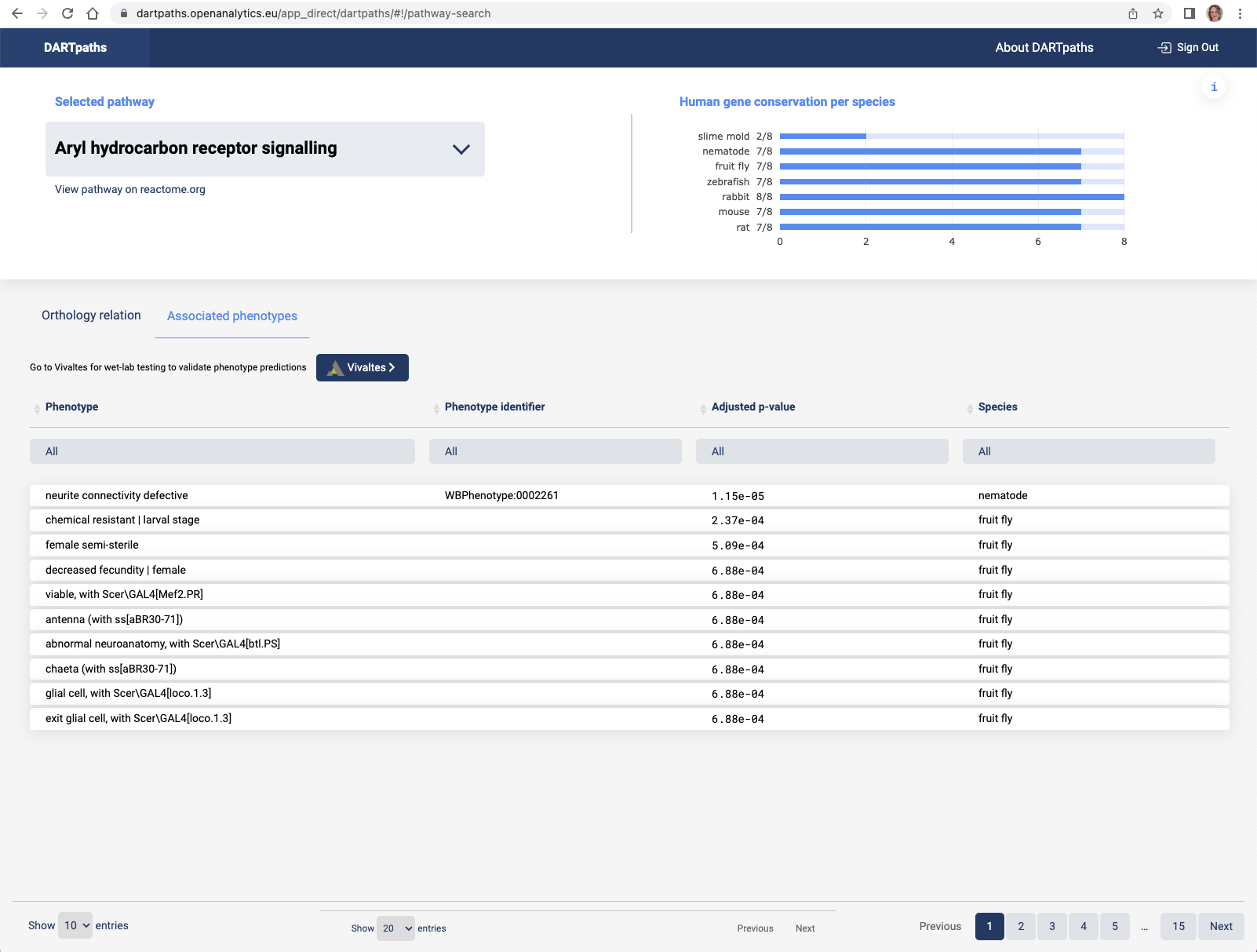


**Figure S21.** Associated phenotypes in pathway exploration page.

*Note.* Associated phenotypes are predicted with the pathway to phenotype prediction algorithm based on genetic variation induced phenotypes of gene members of the pathway. Precomputed enrichment values are present in the application. These can be used to predict expected endpoints for interference with this pathway in model organisms.


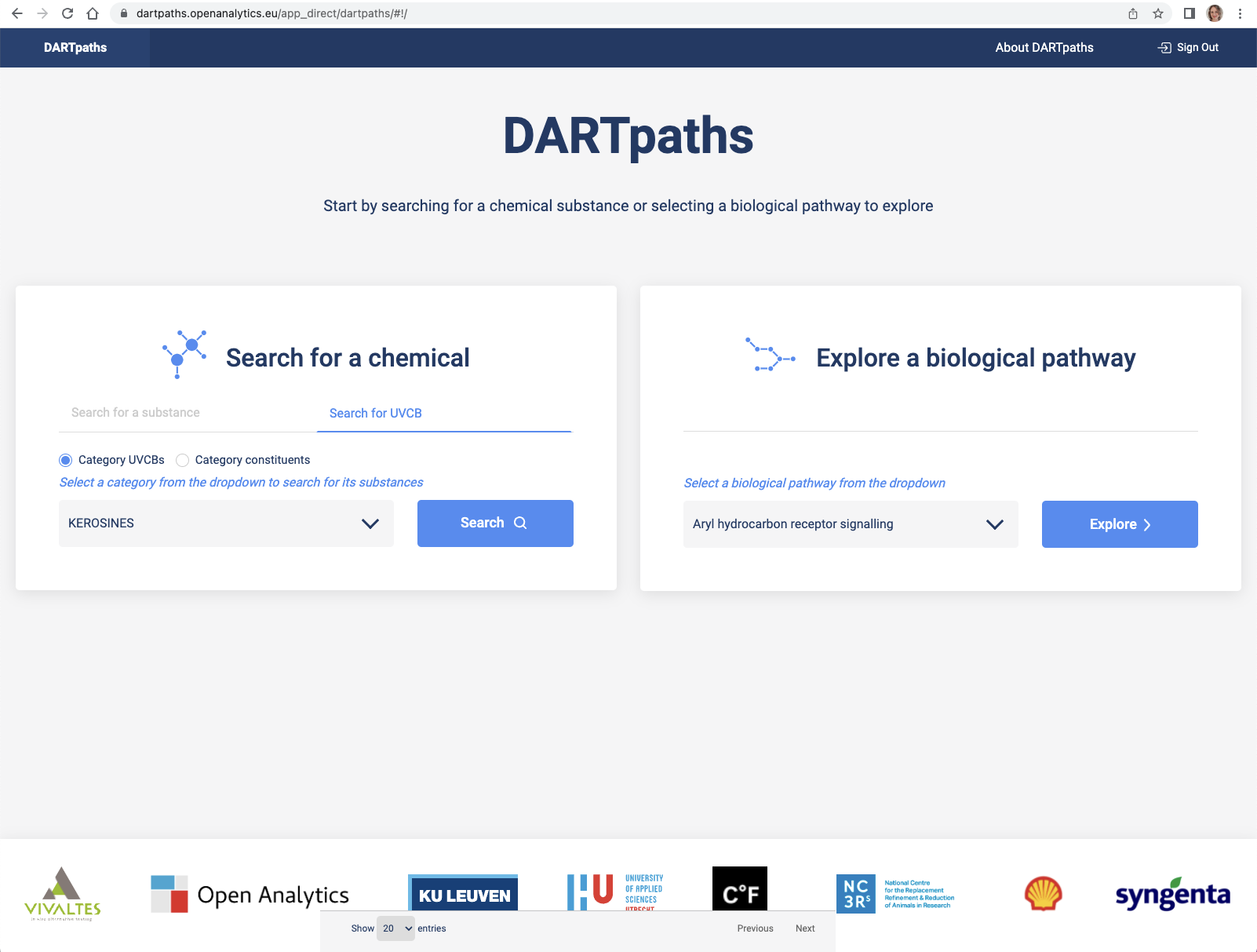


**Figure S22.** UVCB exploration.

*Note.* Also complex compounds are found in the application.


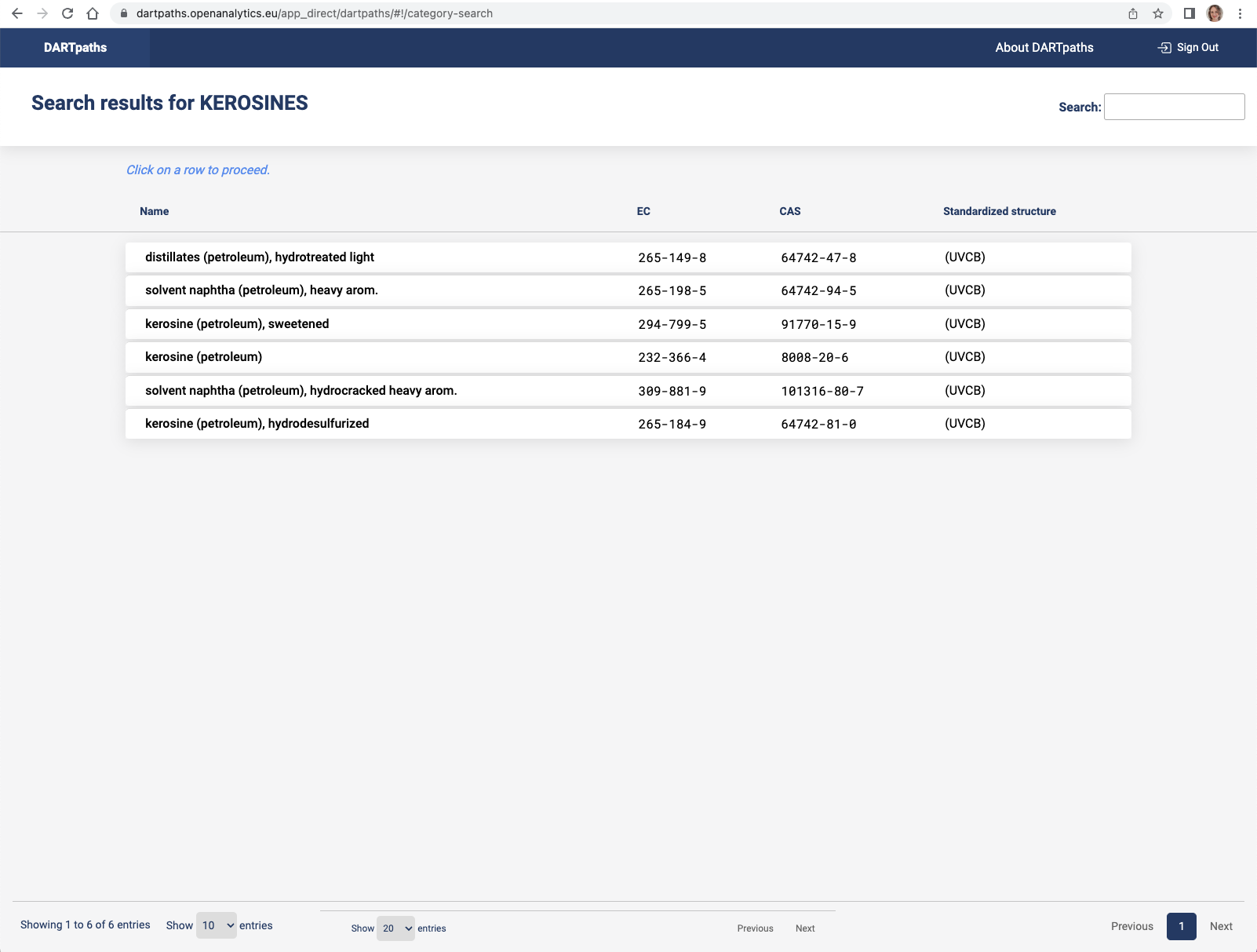


**Figure S23.** Kerosines search results.


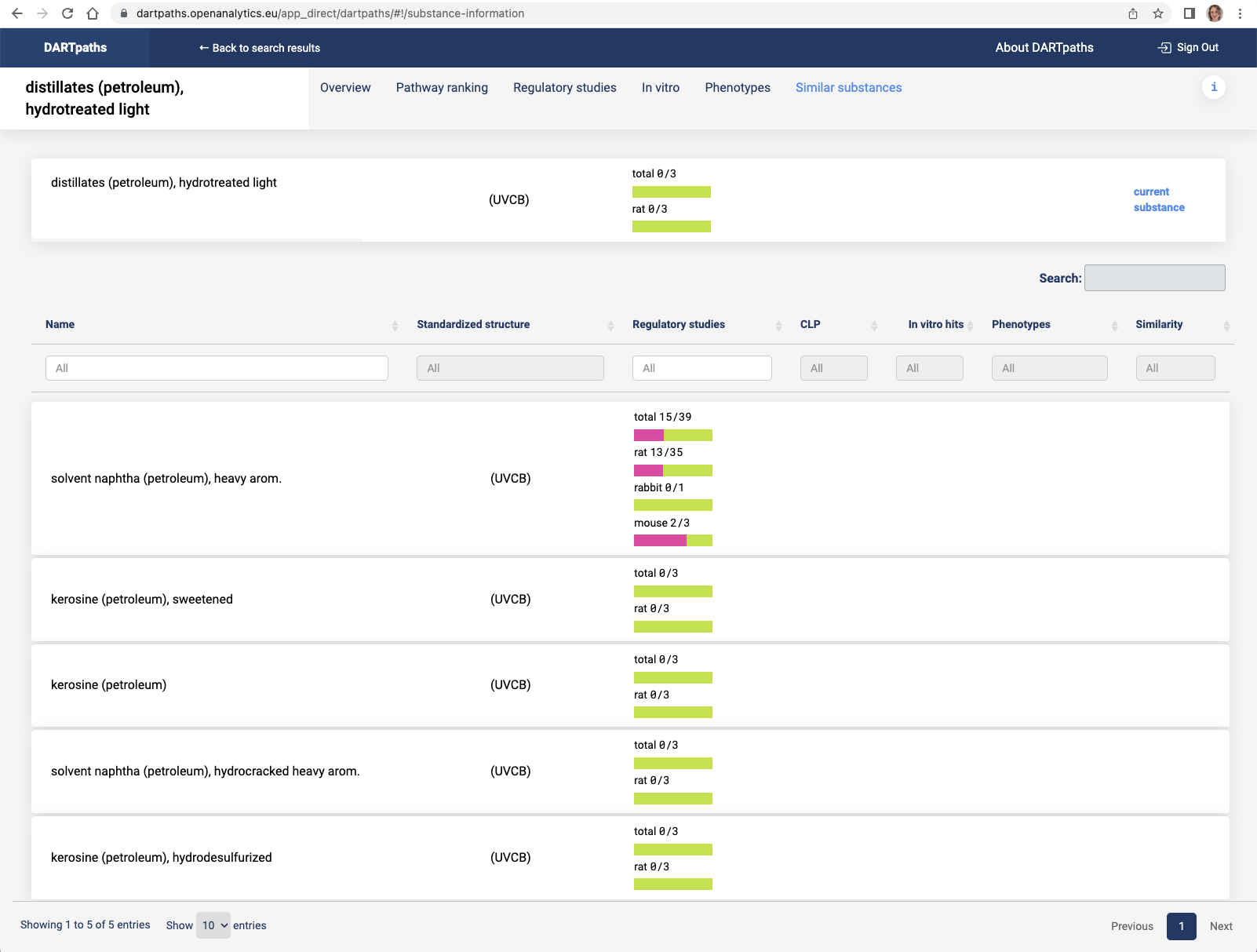


**Figure S24.** Similar substances of the selected UVCB.

*Note*. Through the tab similar substances, compounds that compose the UVCB are found together with regulatory information, in vitro studies etc.


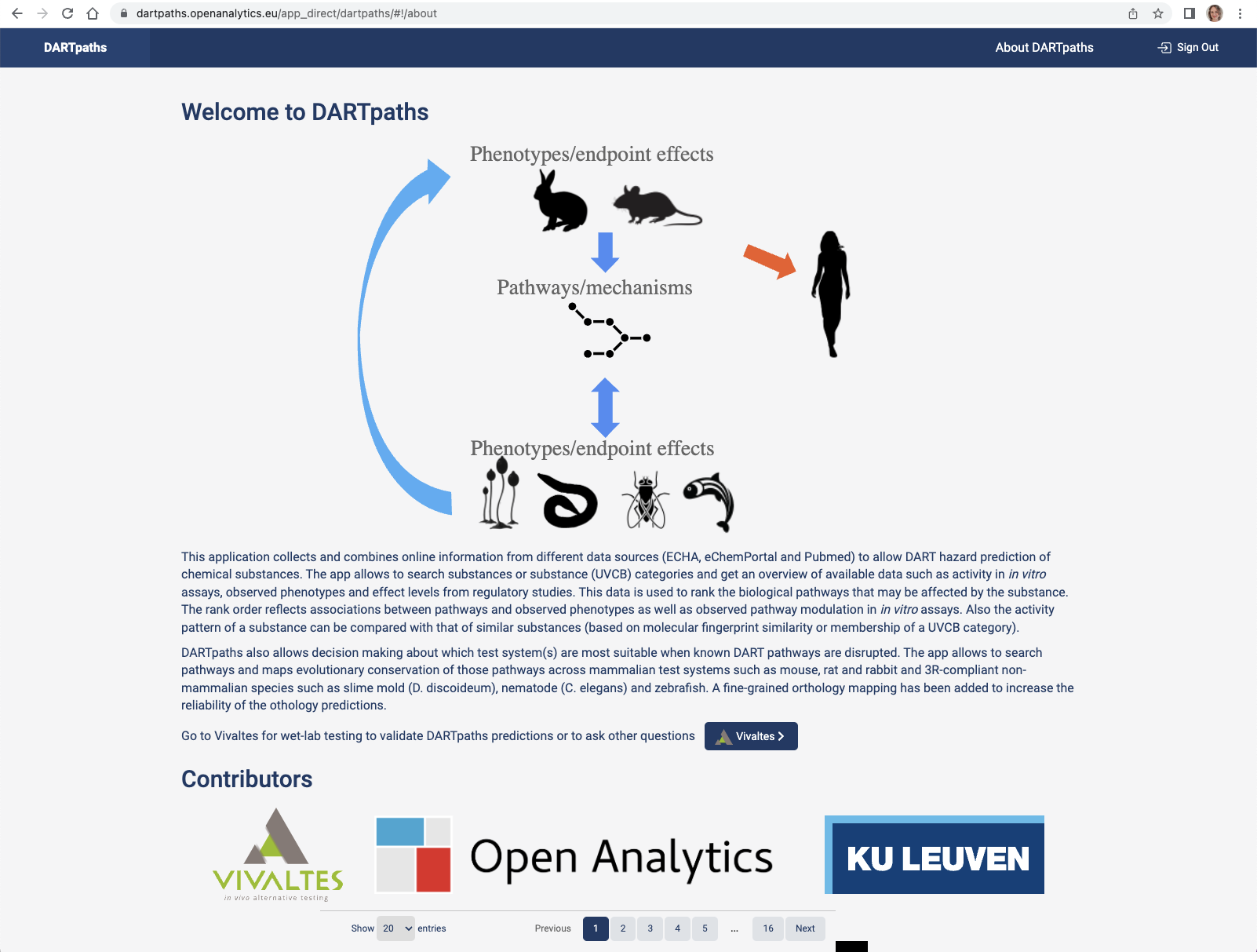


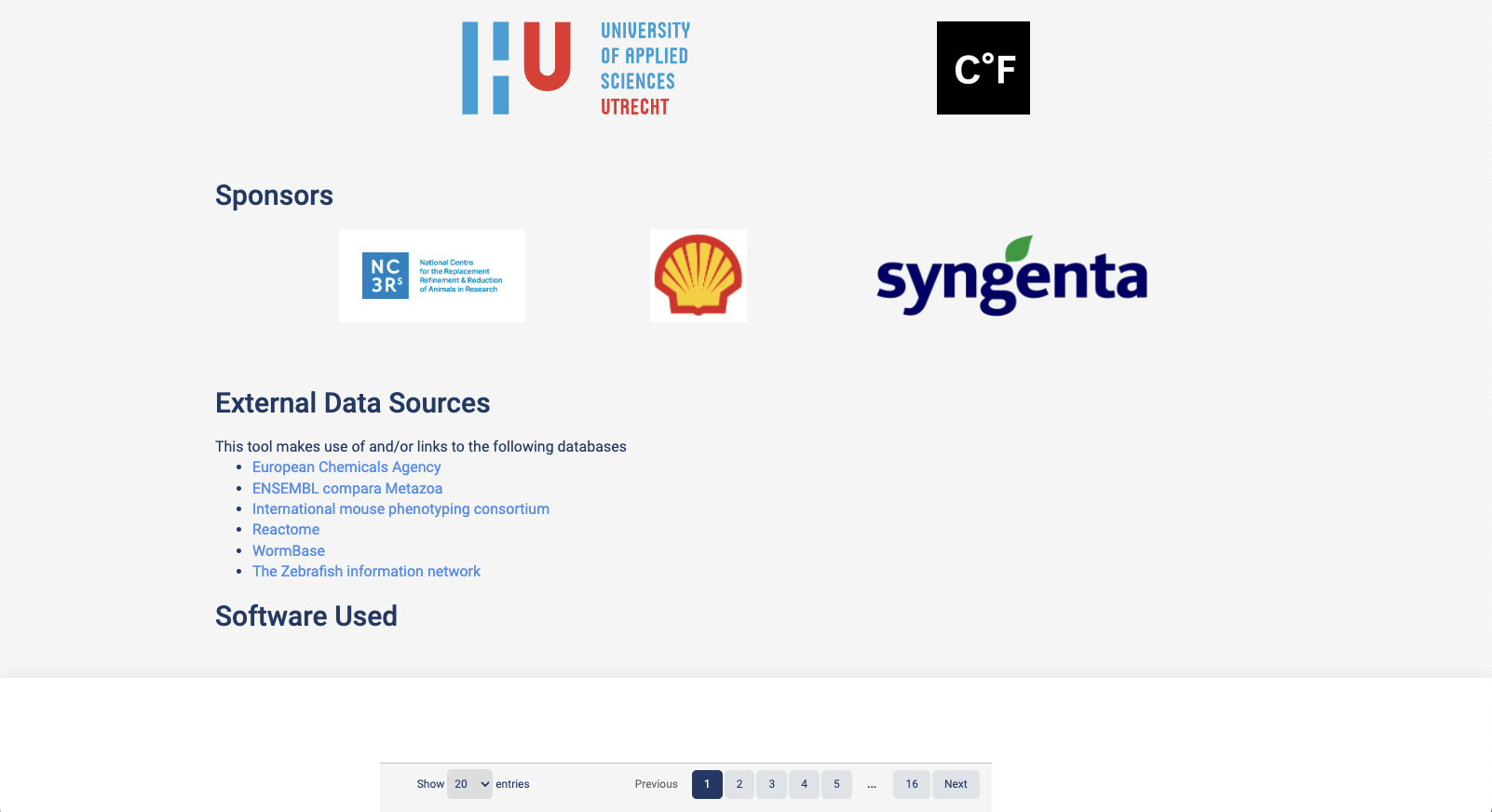


**Figure S25.** The about page of DARTpaths.

# Case study

The goal of this example case study is to identify pathways underlying phenotypes observed as a result of exposure to Diethylstilbestrol (DES). DES is a non-steroidal estrogen that was previously used to support women during pregnancy who had a history of recurrent miscarriages. However, daughters exposed to DES during early development *in utero*, would develop clear-cell carcinoma later in life (Veurink et al, 2005)

First, the phenothypes were extracted from literature and databases (Table S2), and pathways were predicted based on these phenotypes. The phenotypes are quite distinct for nematodes, where mainly aberrations on the cellular level are observed and zebrafish where aberrations in heart development are seen, versus the mammalian phenotypes that include tumore incidence.

Predicted pathways for non-mammalian and mammalian phenotypes as well as *in vitro* targets were combined using the Phenotype to Pathway Enrichment Analysis (Table S3).

**Table S2**. Phenotypes linked to DES in Dartpaths

| **species** | **phenotypeid** | **phenotypename** |
| --- | --- | --- |
| mammalian | MP:0001260 | increased body weight |
| mammalian | MP:0001785 | edema |
| mammalian | MP:0004904 | increased uterus weight |
| mammalian | MP:0003674 | oxidative stress |
| mammalian | MP:0006042 | increased apoptosis |
| mammalian | MP:0000352 | decreased cell proliferation |
| mammalian | MP:0002080 | prenatal lethality |
| mammalian | MP:0009674 | decreased birth weight |
| mammalian | MP:0011940 | decreased food intake |
| mammalian | MP:0003953 | abnormal hormone level |
| mammalian | MP:0001156 | abnormal spermatogenesis |
| mammalian | MP:0002685 | abnormal spermatogonia proliferation |
| mammalian | MP:0005250 | decreased Sertoli cell number |
| mammalian | MP:0005536 | decreased Leydig cell number |
| mammalian | MP:0001922 | reduced male fertility |
| mammalian | MP:0010299 | increased mammary gland tumor incidence |
| mammalian | MP:0004244 | abnormal miscarriage rate |
| mammalian | MP:0002294 | short gestation period |
| mammalian | MP:0002081 | perinatal lethality |
| mammalian | MP:0014052 | increased male germ cell apoptosis |
| zebrafish | ZP:0011289 | heart development process quality, abnormal |
| zebrafish | ZP:0018025 | thyroid gland development process quality, abnormal |
| zebrafish | ZP:0005415 | cardiac ventricle morphology, abnormal |
| zebrafish | ZP:0004978 | cardiac ventricle shape, abnormal |
| zebrafish | ZP:0001589 | embryo development disrupted, abnormal |
| c. elegans | WBPhenotype:0001171 | shortened life span |
| c. elegans | WBPhenotype:0001028 | nuclear appearance variant |
| c. elegans | WBPhenotype:0001813 | synapsis defective |

Although it is now well known that exposure to DES can lead to various adverse outcomes, in this use case we show that using pathway prediction, researchers can generate hypotheses and study interconnected pathways that are not obvious from currently available data and find underlying Adverse Outcome Pathways.

When the pathway enrichment analysis of DES was performed with DARTpaths, the following pathways were among the list of significantly enriched pathways (FDR-corrected *p* < 0.05).

**Table S3**. Predicted pathways for DES.

| **Reactome pathway stable identifier** | **Pathway name** | **In vitro** | **Score In Vitro** | **Adjusted p-value** | **Adjusted p-value mammalian** | **Adusted p-value NAM** |
| --- | --- | --- | --- | --- | --- | --- |
| R-HSA-8978934 | Metabolism of cofactors | 0 | 0 | 0.0272 | 0.00992 | 0.0412 |
| R-HSA-8957322 | Metabolism of steroids | 2 | 0.05 | 0.0272 | 0.0141 | 0.0412 |
| R-HSA-8978868 | Fatty acid metabolism | 0 | 0 | 0.0272 | 0.00992 | 0.0412 |
| R-HSA-1369007 | Mitochondrial ABC transporters | 0 | 0 | 0.0272 | 0.00992 | 0.0412 |
| R-HSA-190236 | Signaling by FGFR | 2 | 0.048 | 0.0272 | 0.0141 | 0.0412 |
| R-HSA-170834 | Signaling by TGF-beta Receptor Complex | 0 | 0 | 0.0272 | 0.00992 | 0.0412 |
| R-HSA-9711123 | Cellular response to chemical stress | 0 | 0 | 0.0272 | 0.00992 | 0.0412 |
| R-HSA-157579 | Telomere Maintenance | 0 | 0 | 0.0272 | 0.00992 | 0.0412 |
| R-HSA-110314 | Recognition of DNA damage by PCNA-containing replication complex | 0 | 0 | 0.0272 | 0.00992 | 0.0412 |
| R-HSA-9663891 | Selective autophagy | 0 | 0 | 0.0272 | 0.00992 | 0.0412 |

Even though the phenotypes are distinct, the molecular pathways predicted by mammalian phenotypes overlap with the pathways predicted by *in vitro* targets and non-mammalian phenotypes. This demonstrates that the cross-species integration of pathways predicted from non-mammalian phenotypes and *in vitro* data has added value for mammalian Adverse Outcome Pathway prediction.

It is clear that metabolism of steroids is one of the pathways that is associated with similar phenotypes as exposure to DES. Not only the Estrogen Receptor is bound by DES, also the enzymes involved in the metabolism of steroid hormones such as CYP19A that catalyzes the conversion of C19 androgens to C18 estrogens and TSPO that promotes the transport of cholesterol across mitochondrial membranes and may play a role in lipid metabolism.

The genetic variants in the pathway ‘Metabolism of steroids’ have the same phenotypes as exposure to DES both in mammals and in non-mammalian New Approch Methodology (NAM) model organisms zebrafish and the nematode *C. elegans*.

Extracted from (Kuhl. 2009):

*“ The classical estrogenic hormones are C18 steroids with a phenolic structure in ring A. …*

*There are also non-steroidal estrogens like* ***diethylstilbestrol*** *and its derivatives.* ***tamoxifen.*** *clomiphene. raloxifene. etc.. and the various phytoestrogens.* ***Their structure allows binding to the Estrogen Receptors (ERs)****, but – as they do not ﬁt exactly in the ligand-binding domain of the receptor protein – prevents the correct interaction necessary for exerting the same biological effects as estradiol. These structure-dependent peculiarities and the existence of two different ERs are the basis of the development of selective estrogen receptor modulators (SERMs). The prerequisite of steroid-like actions of non-steroidal compounds is a stiff, planar structure which is brought about by socalled conjugate double bonds (single bond alternating with double bond) which couple the aromatic systems of two rings.* ***Diethylstilbestrol is a potent estrogen,*** *because it is a stiff plane with two hydroxy groups in a distance similar to that of the estradiol molecule. Compounds with no phenolic hydroxy group (e.g. tamoxifen) are prodrugs that are converted to the active estrogen agonist or antagonist (e.g. 4-hydroxy-tamoxifen) in the intestinal tract and liver.”*

In addition signaling by FGFR pathway seems to play a role in the toxicity caused by DES as genetic variants of this pathway also show similar phenotypes and two targets are part of the FGFR signaling pathway, FGFR1 and PIK3CA. PIK3CA mutations have been found in breast cancer (Zardavas et al, 2014). Thus the link between DES and cancer could be via disrupted signaling through PIK3CA.

Other reports have found increased levels of FGFR signaling proteins in mouse neoDES vagina’s compared to controls, suggesting that indeed this signaling pathway also plays a role in the adverse outcomes (Miyagawa et al, 2004).

# References

Benjamini. Y.. Hochberg. Y. (1995) Controlling the False Discovery Rate: A Practical and Powerful Approach to Multiple Testing. Journal of the Royal Statistical Society: Series B (Methodological). 57(1). 289-300. doi: 10.1111/j.2517-6161.1995.tb02031.x.

Benjamini, Y., Heller, R., Yekutieli, D., (2009) Selective inference in complex research. Philosophical transactions of the royal society A. **367:** 4255–4271

Kuhl.H. (2009) Pharmacology of estrogens and progestogens: influence of different routes of administration. Climacteric. 8. 3–63.

Miyagawa, S., Suzuki, A., Katsu, Y., Kobayashi, M., Goto, M., Handa, H., Watanabe, H., Iguchi, T. (2004) Persistent gene expression in mouse vagina exposed neonatally to diethylstilbestrol. J Mol Endocrinol 32: 663-77.

Veurink, M., Koster, M., de Jong-van den Berg L.T.W. (2005) The history of DES, lessons to be learned. Pharm World Sci 27. 139-43.

Wilson. Daniel J. (2020) Generalized Mean P-Values for Combining Dependent Tests: Comparison of Generalized Central Limit Theorem and Robust Risk Analysis. Wellcome Open Research 5(March): 55. doi: 10.12688/wellcomeopenres.15761.1.

Zardavas, D., Wayne, A.P., Loi, S. (2014) PIK3CA mutations in breast cancer: reconciling findings from preclinical and clinical data. Breast Cancer Research 16: 201
